# Supplementary material for: A single-cell transcriptional gradient in human cutaneous memory T cells restricts Th17/Tc17 identity
Source: Cell Rep Med. 2022 Aug 16;3(8):100715. doi: 10.1016/j.xcrm.2022.100715 (PMC9418858; doi:10.1016/j.xcrm.2022.100715)
Supplement: Document S1. Figures S1–S6 [file mmc1.pdf]

Cell Reports Medicine, Volume *3*

Supplemental information

A single-cell transcriptional gradient in human cutaneous memory T cells restricts Th17/Tc17 identity

Christopher P. Cook, Mark Taylor, Yale Liu, Ralf Schmidt, Andrew Sedgewick, Esther Kim, Ashley Hailer, Jeffrey P. North, Paymann Harirchian, Hao Wang, Sakeen W. Kashem, Yanhong Shou, Timothy C. McCalmont, Stephen C. Benz, Jaehyuk Choi, Elizabeth Purdom, Alexander Marson, Silvia B.V. Ramos, Jeffrey B. Cheng, and Raymond J. Cho

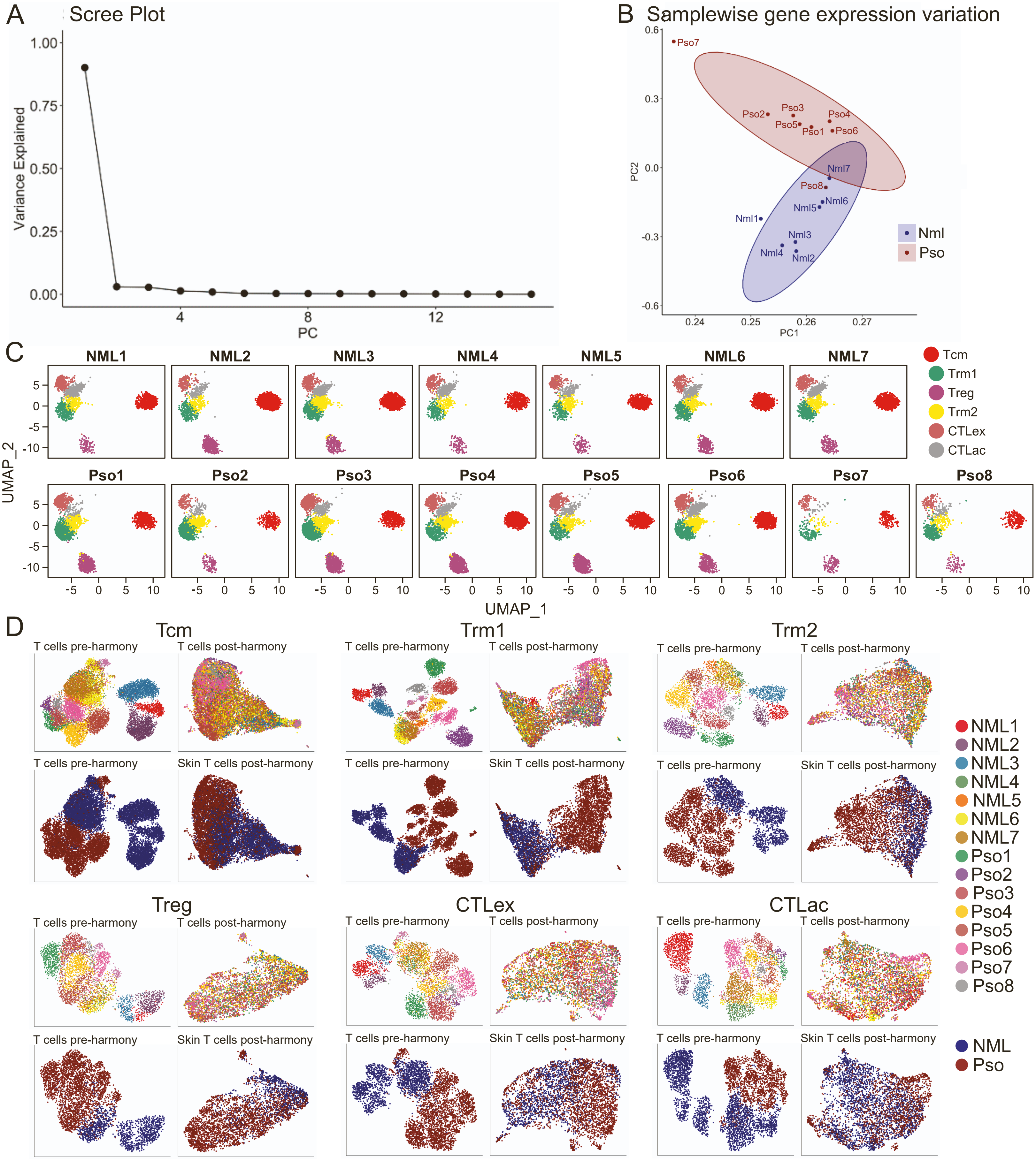

Figure S1 - Illustration of biological data consistency across patient samples (Related to Figure 1). A) Scree plot of eigendecomposition showing that PC1 and PC2 explain 93.4% of variation in gene expression (all 15 samples). B) Plot of all samples vs. PCs 1 and 2 illustrating that normal (Nml) and psoriasis (Pso) samples cluster coherently in PC space, with sparse overlap in their respective 95%-confidence bivariate ellipses. C) uMAP graphs showing T cell populations from each Nml and Pso sample. While Main Figure 1B shows each cluster derives substantial representation from each healthy and normal sample, Figure S1C illustrates how the distinct identity of each cluster is broadly replicated in each sample. D) uMAP representations of each major T cell cluster before and after batch correction (all 15 samples aggregated), illustrating necessity for correction.

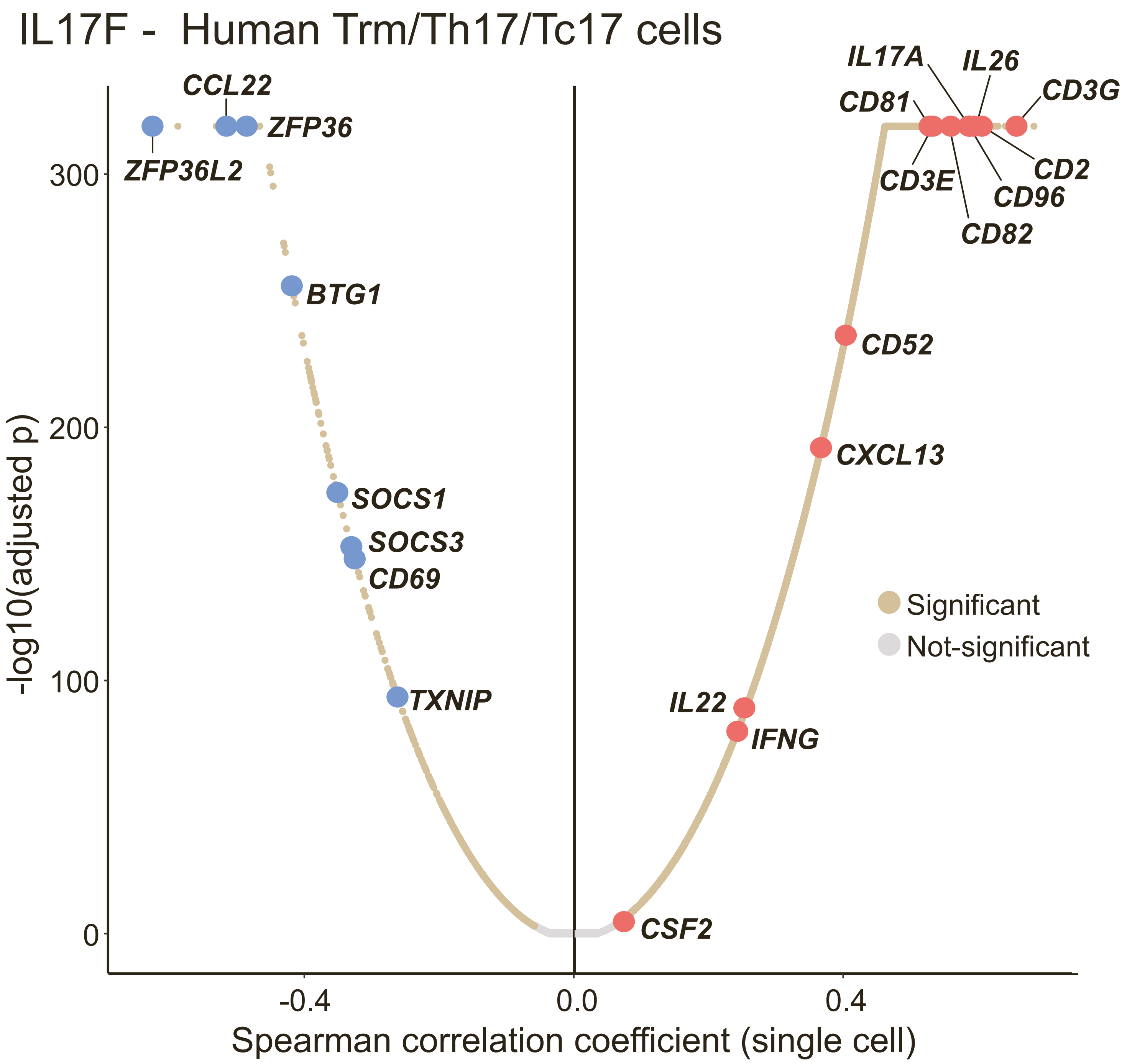

Figure S2 - Enrichment in *IL17F*-expressing single skin Trm1 cells for expression of pro-inflammatory transcripts and cytokines (Related to Figure 2). Positive correlation of *IL17F* expression in single Trm1 cells with expression of pro-inflammatory transcripts and cytokines (right half of graph, labelled in red). Deficiency in *IL17F*-expressing single Trm1 cells of inflammation-suppressive genes such as *ZFP36L2*, *ZFP36*, and *BTG1* is shown as negative Spearman correlations (left half of graph, labelled in blue). Data is shown for all 8 psoriasis and 7 normal samples.

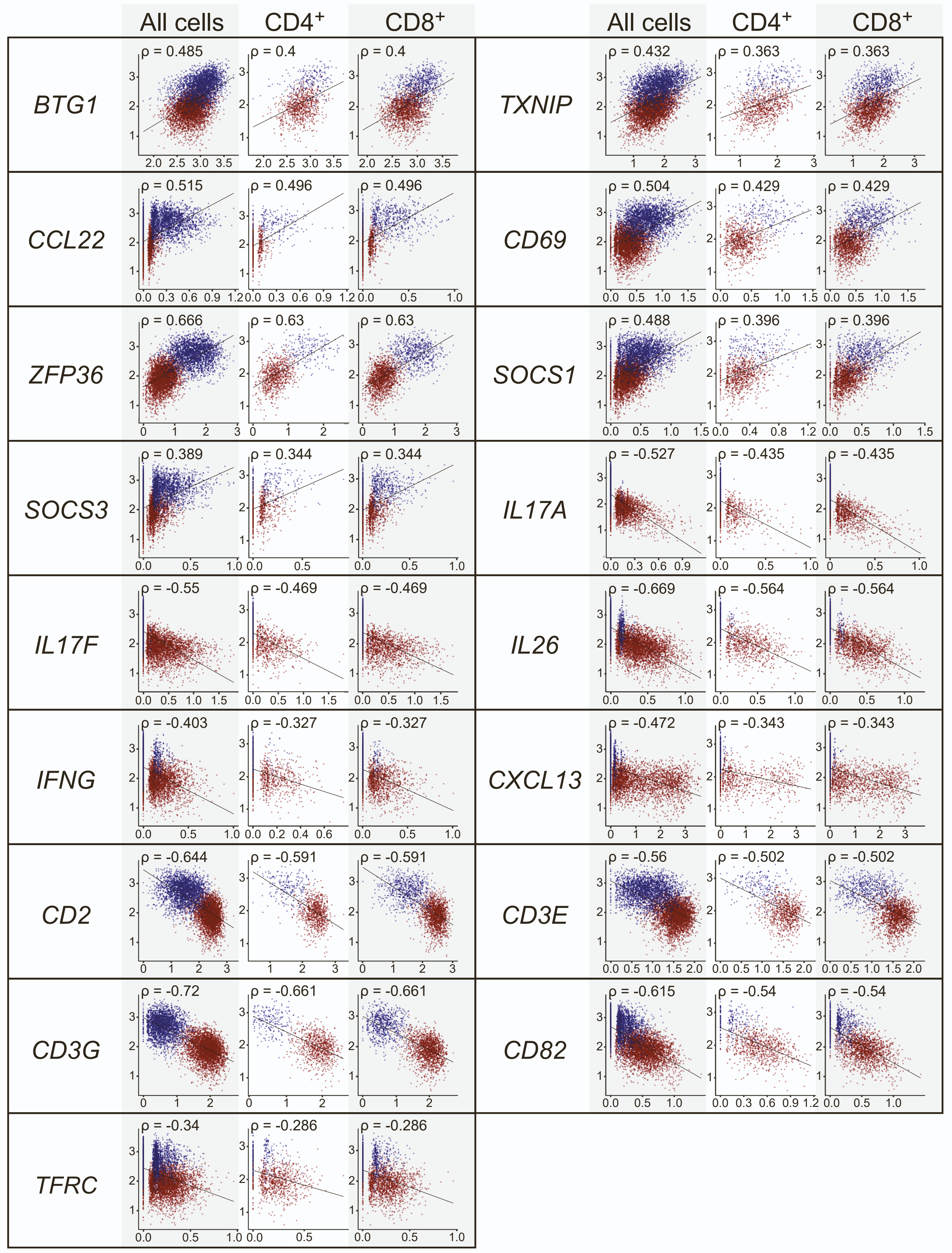

Figure S3 – *ZIST* gradient is detected in both Th17 and Tc17 subpopulations (Related to Figure 2). *ZFP36L2* positive and negative correlations with *ZIST* genes and Th17/Tc17 cytokines are shown to persist in both CD4+ (i.e. Th17) and CD8+ (i.e. Tc17) single cell subpopulations when separated based on expression of one or the other transcript (double negative and double positive cells were excluded, red shows all 8 psoriasis samples pooled and blue shows all 7 healthy controls pooled). y-axis shows *ZFP36L2* expression, x-axis shows imputed cytokine transcript levels, each point represents a single skin-resident T cell.

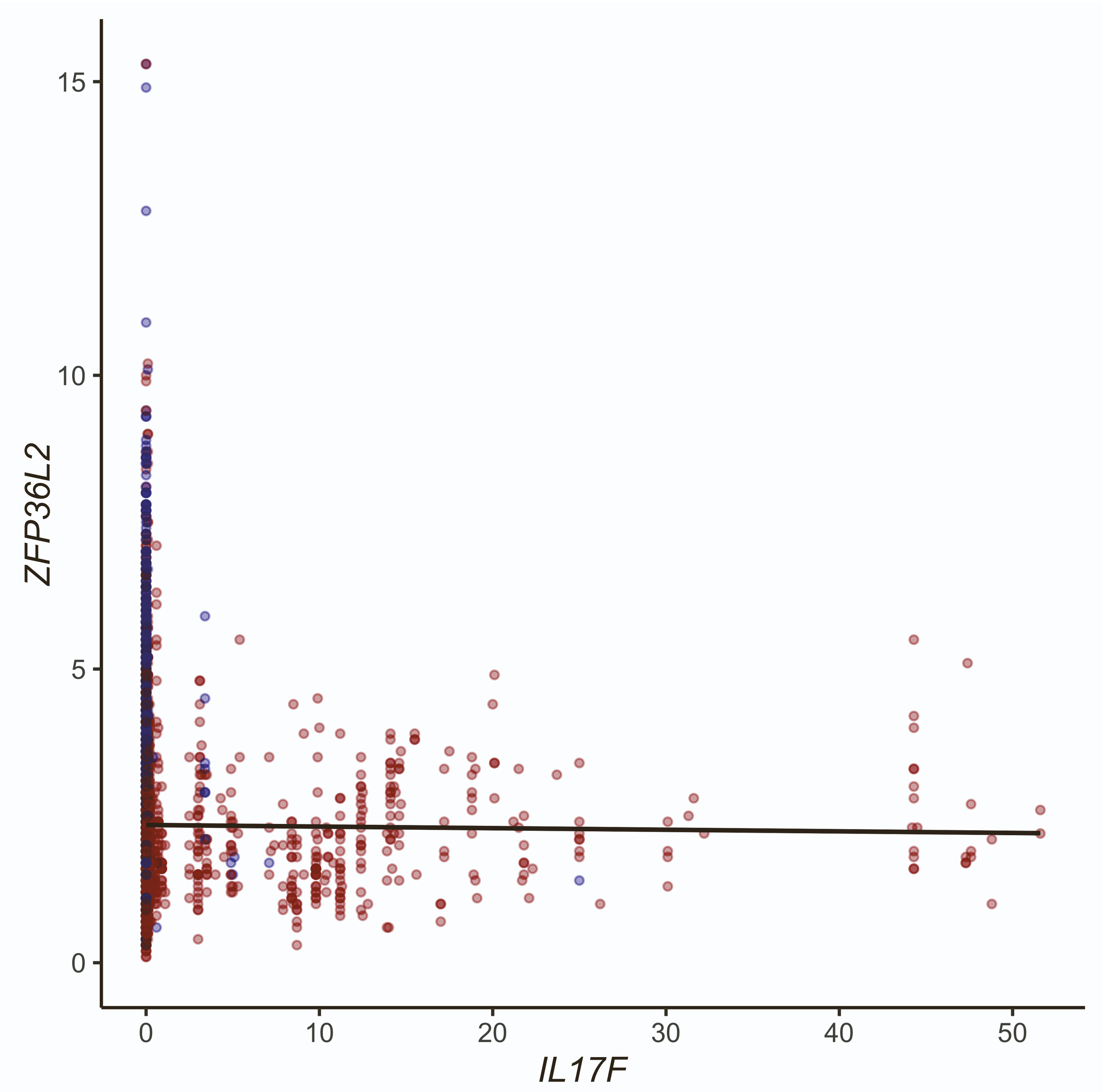

Figure S4 - Suppression of *IL17F* in *ZFP36L2*-expressing human CD4+ PBMCs (Related to Figures 2, 4). Relationship between *IL17F* (x-axis) and *ZFP36L2* (y-axis) expression in CD3/CD28 bead-activated (red) and naïve (blue) T cells from Cano-Gamez *et al*, imputed transcript levels shown, each point represents a single T cell.

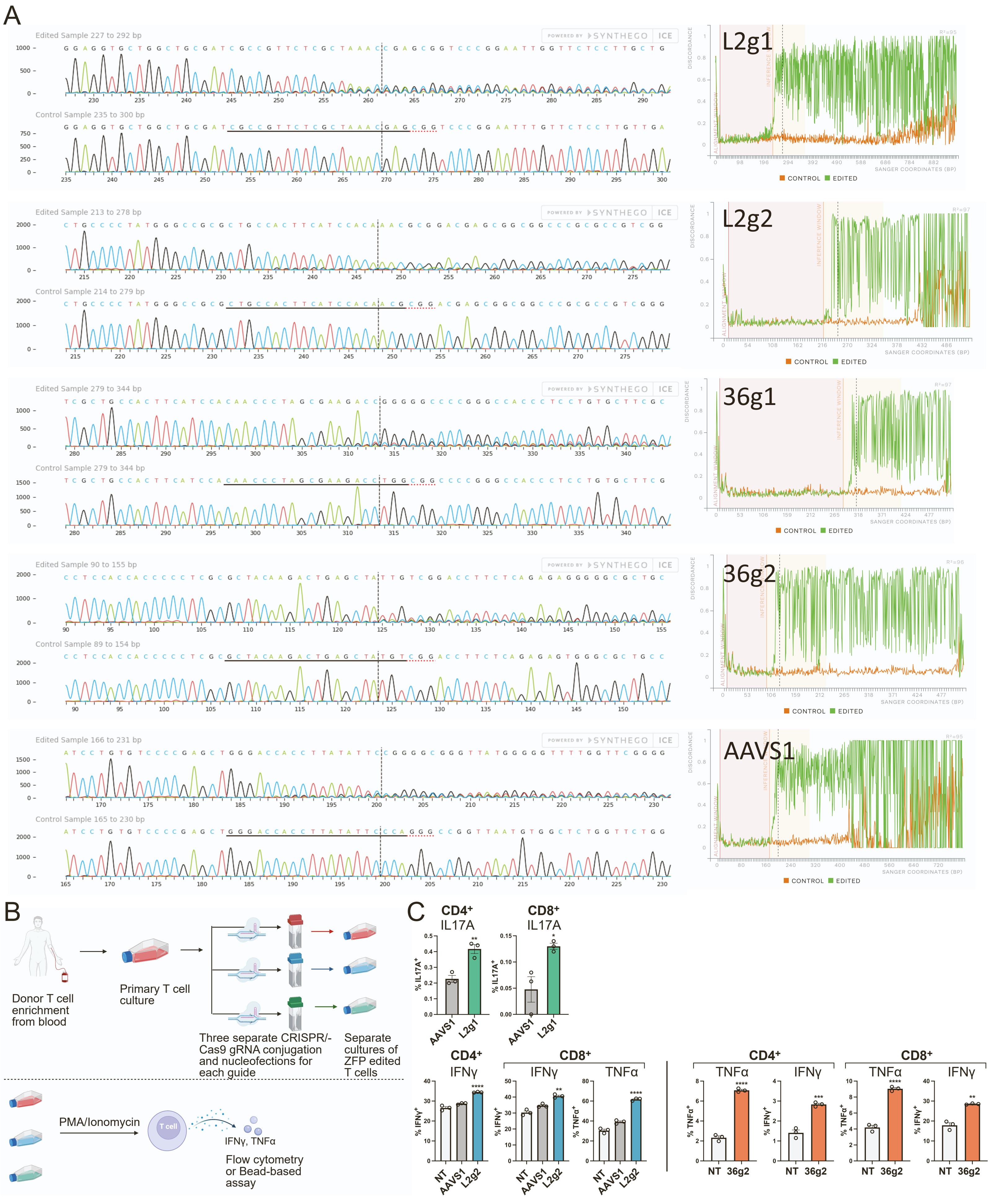

Figure S5 - Sanger sequencing details on CRISPR/Cas9 guides and additional flow cytometry data for a distinct donor (Related to Figure 3). A) Representative Sanger traces confirming CRISPR/Cas9 knockdown of guides used for Figure 3. B) Schematic showing CRISPR/Cas9 knockout strategy in Figure 3. C) Additional CRISPR/Cas9 knockout experiments and intracellular cytokine staining results for IL17A (2nd guide displayed), IFNγ and TNFα in *ZFP36L2* knockout cells, and IFNγ and TNFα in *ZFP36* knockout cells.

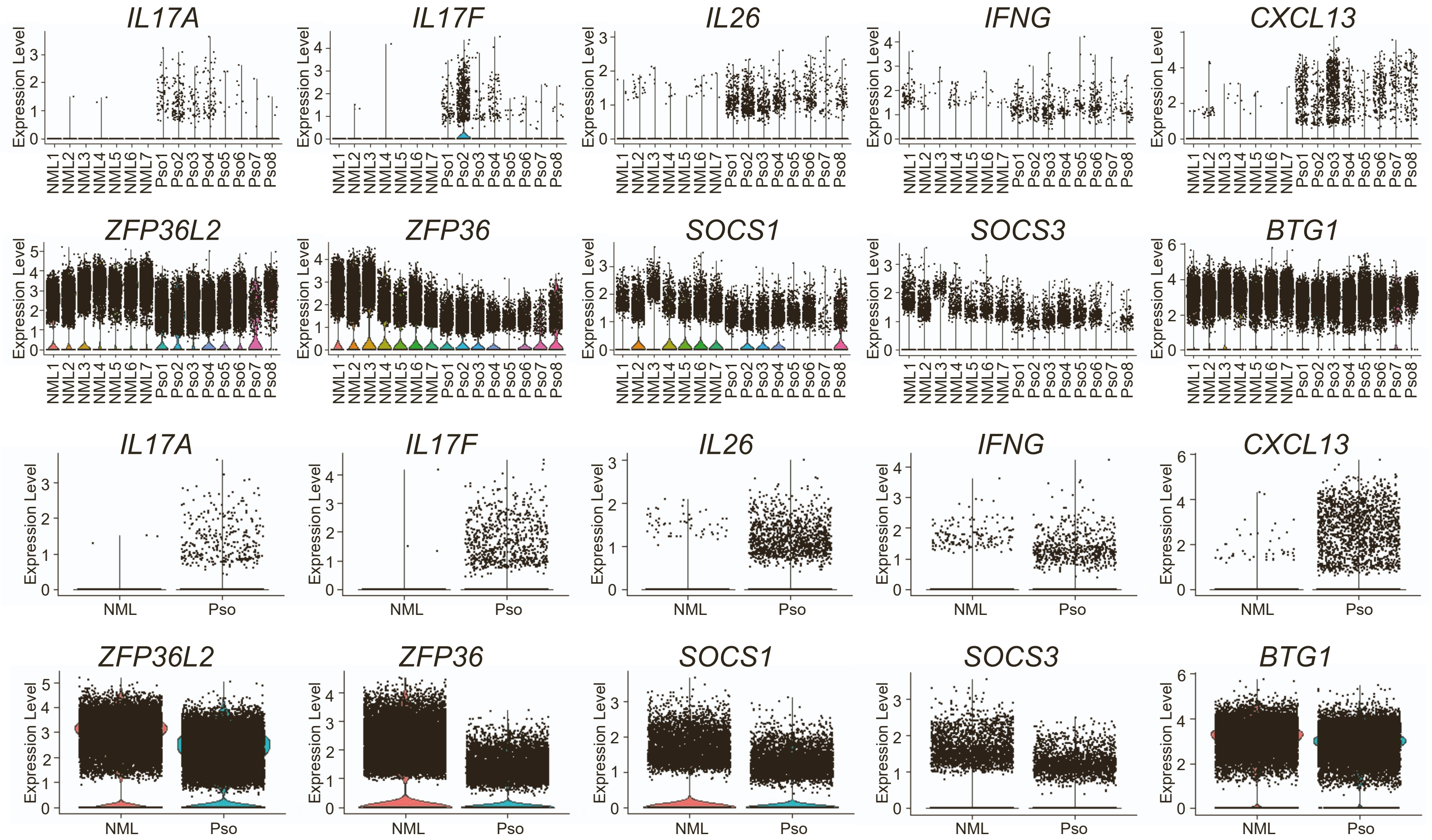

Figure S6 – Individual and aggregate patient expression of representative *ZIST* and Th17/Tc17 genes, showing sample-level consistency in DEG patterns (Related to Figure 5). Violin expression plots demonstrating consistency of expression changes in key *ZIST* and Th17/Tc17 inflammatory transcripts across individual and aggregated samples.
