# Supplementary material for: A single-cell transcriptional gradient in human cutaneous memory T cells restricts Th17/Tc17 identity
Source: Cell Rep Med. 2022 Aug 16;3(8):100715. doi: 10.1016/j.xcrm.2022.100715 (PMC9418858; doi:10.1016/j.xcrm.2022.100715)
Supplement: Document S2. Article plus supplemental information [file mmc11.pdf]

Cell Reports
Medicine

Article

A single-cell transcriptional gradient in human cutaneous memory T cells restricts Th17/Tc17 identity

Graphical abstract

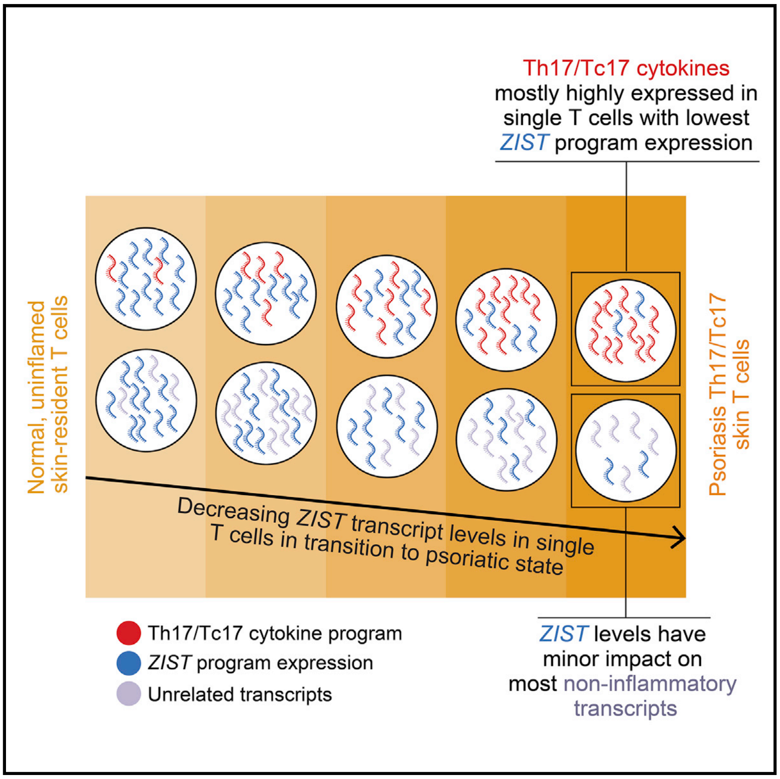

Highlights

- Psoriatic Th17/Tc17 cells dampen an inflammation-suppressive program (*ZIST*)
- Knockout of *ZIST* transcripts *ZFP36L2*, *ZFP36* in T cells induces Th17/Tc17 cytokines
- IL23 blockade fails to normalize attenuated *ZIST* levels in healed psoriasis lesions

Authors

Christopher P. Cook, Mark Taylor, Yale Liu, ..., Silvia B.V. Ramos, Jeffrey B. Cheng, Raymond J. Cho

Correspondence

Jeffrey.Cheng@ucsf.edu (J.B.C.),
raymond.cho@ucsf.edu (R.J.C.)

In brief

Cook et al. show that Th17/Tc17 expression occurs in a gradient within psoriatic single T cells, paralleled by attenuation of an inflammation-suppressive gene-expression program termed *ZIST*. Persistent *ZIST* suppression, even in therapeutically improved psoriatic lesions, suggests it contributes to recurrence of disease after treatment withdrawal.

Cook et al., 2022, Cell Reports Medicine *3*, 100715
August 16, 2022 © 2022 The Author(s).
https://doi.org/10.1016/j.xcrm.2022.100715

CellPress

Cell Reports Medicine

CellPress OPEN ACCESS

Article

A single-cell transcriptional gradient in human cutaneous memory T cells restricts Th17/Tc17 identity

Christopher P. Cook,[1,2,14] Mark Taylor,[1,3,14] Yale Liu,[2,4,14] Ralf Schmidt,[5] Andrew Sedgewick,[6] Esther Kim,[7] Ashley Hailer,[1] Jeffrey P. North,[1] Paymann Harirchian,[1,2] Hao Wang,[8] Sakeen W. Kashem,[1,2] Yanhong Shou,[9] Timothy C. McCalmont,[1,10,11] Stephen C. Benz,[6] Jaehyuk Choi,[12] Elizabeth Purdom,[8] Alexander Marson,[5] Silvia B.V. Ramos,[13] Jeffrey B. Cheng,[1,2,15,*] and Raymond J. Cho[1,15,16,*]

[1]Department of Dermatology, University of California, San Francisco, San Francisco, CA, USA
[2]Dermatology, Veterans Affairs Medical Center, San Francisco, CA, USA
[3]Clinical Research Centre, Medical University of Białystok, Białystok, Poland
[4]Department of Dermatology, The Second Affiliated Hospital of Xi'an Jiaotong University, Xi'an, ShaanXi 710004, P.R. China
[5]Gladstone-UCSF Institute of Genomic Immunology, San Francisco, CA, USA
[6]NantHealth, Inc, El Segundo, CA, USA
[7]Division of Plastic Surgery, University of California, San Francisco, San Francisco, CA, USA
[8]Department of Statistics, University of California, Berkeley, Berkeley, CA, USA
[9]Department of Dermatology, Huashan Hospital, Fudan University, Shanghai, P.R. China
[10]Department of Pathology, University of California, San Francisco, San Francisco, CA, USA
[11]Golden State Dermatology Associates, Walnut Creek, CA, USA
[12]Department of Dermatology, Northwestern University, Evanston, IL, USA
[13]Department of Biochemistry and Biophysics, University of North Carolina at Chapel Hill, Chapel Hill, NC, USA
[14]These authors contributed equally
[15]Senior author
[16]Lead contact
*Correspondence: Jeffrey.Cheng@ucsf.edu (J.B.C.), raymond.cho@ucsf.edu (R.J.C.)
https://doi.org/10.1016/j.xcrm.2022.100715

SUMMARY

The homeostatic mechanisms that fail to restrain chronic tissue inflammation in diseases, such as psoriasis vulgaris, remain incompletely understood. We profiled transcriptomes and epitopes of single psoriatic and normal skin-resident T cells, revealing a gradated transcriptional program of coordinately regulated inflammation-suppressive genes. This program, which is sharply suppressed in lesional skin, strikingly restricts Th17/Tc17 cytokine and other inflammatory mediators on the single-cell level. CRISPR-based deactivation of two core components of this inflammation-suppressive program, ZFP36L2 and ZFP36, replicates the interleukin-17A (IL-17A), granulocyte macrophage-colony-stimulating factor (GM-CSF), and interferon gamma (IFNγ) elevation in psoriatic memory T cells deficient in these transcripts, functionally validating their influence. Combinatoric expression analysis indicates the suppression of specific inflammatory mediators by individual program members. Finally, we find that therapeutic IL-23 blockade reduces Th17/Tc17 cell frequency in lesional skin but fails to normalize this inflammatory-suppressive program, suggesting how treated lesions may be primed for recurrence after withdrawal of treatment.

INTRODUCTION

Cytokine signaling drives common types of pathologic skin inflammation, such as the Th1 and Th17 populations causative for psoriasis vulgaris.[1] Over the past 10 years, targeted inhibition of these pathways has generated effective, biologic therapies for diseases such as psoriasis and revealed skin-resident T cell populations that support recurrent disease.[2] However, many mechanistic aspects of tissue inflammation remain unclear, particularly how pathogenic activation of these pathways is restrained between clinical flares. Nor is it certain how initial inflammatory activation amplifies within immune cell populations to produce symptomatic skin lesions.

The emergence of single-cell RNA sequencing (scRNA-seq) has made it possible to analyze cutaneous biological responses in ever higher resolution.[3–6] These profiles can uniquely reveal inflammatory abnormalities in immune and stromal cells, providing a fine-grained portrait of how pathogenic changes reprogram complex tissues. However, these approaches have not yet closely examined how healthy, uninflamed T cells transition to a pathogenic state in chronic skin disease. In experimental settings outside of skin, T cell populations appear comprised of continuums of cellular identity, which robustly influence inflammatory activation.[7] It is yet unknown if similar gradients operate in clinical disease and how they control pathogenic immunological behavior.

This is an open access article under the CC BY license (http://creativecommons.org/licenses/by/4.0/).

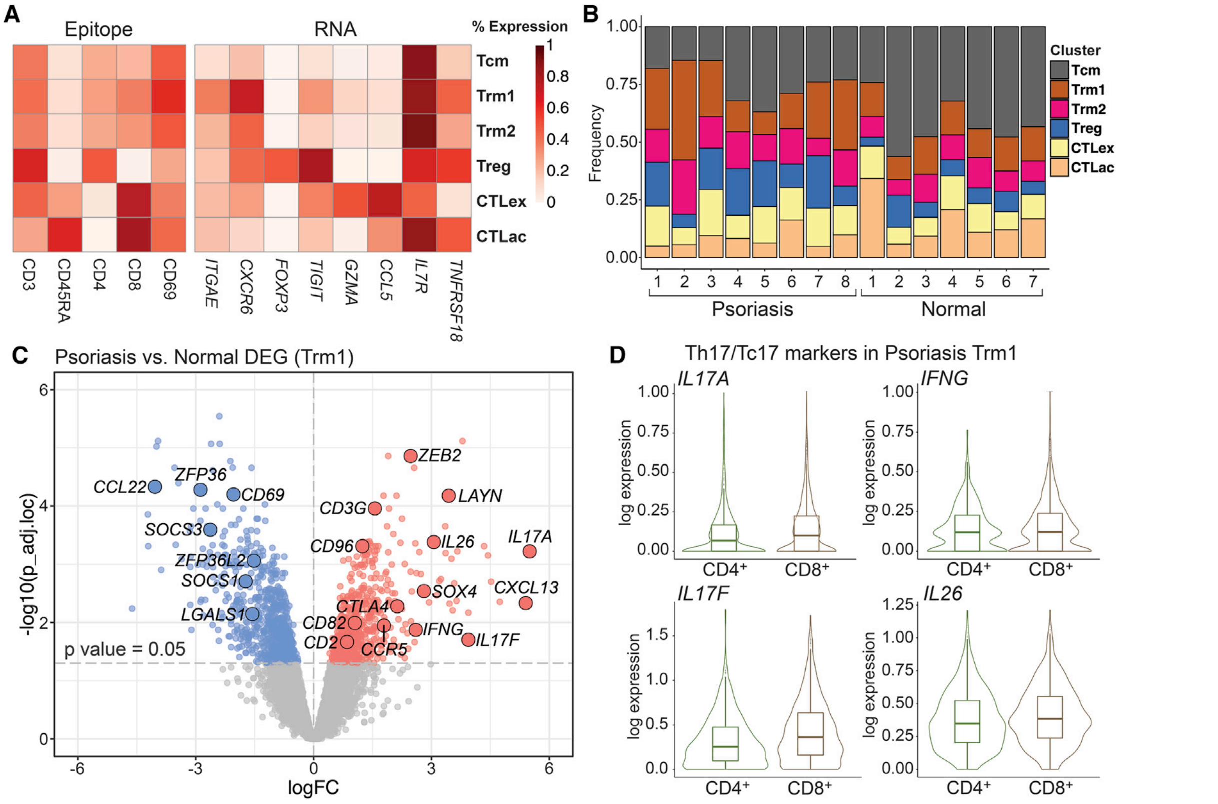

Figure 1. Psoriatic T cells expressing *IL17A* and *IL17F* harbor skin-resident memory markers and classify into both Th17 and Tc17 subpopulations

(A) Single-cell RNA sequencing (scRNA-seq) and cellular indexing of transcriptomes and epitopes sequencing (CITE-seq; protein epitope) marker expression defining major T cell subpopulations (>5% of all T cells) from 8 psoriasis and 7 normal skin samples.

(B) Frequency of six major T cell subpopulations in each donor sample.

(C) Differentially expressed transcripts in the Trm1 subclass in lesional psoriatic skin versus healthy controls (Table S4). The x axis denotes average log2FC in transcript counts between disease and healthy controls (increases in red, decreases in blue). The y axis denotes negative $\log_{10}$ of the adjusted p value. Key cytokines (red) and inflammation-suppressive genes (blue) are labeled.

(D) Psoriatic cytokine expression in both 800 *CD4* transcript-positive cells (Th17) and 1,573 *CD8* transcript-positive cells (Tc17) from 8 psoriasis samples.

To investigate T cell identity and consequence in an *in vivo* setting, we scRNA-seq-profiled $CD45^+$ immune cells isolated from lesional skin in eight patients with active cases of the prototypical Th17/Tc17 skin disease psoriasis vulgaris and compared them with seven uninflamed, normal controls. In addition to searching for molecular abnormalities distinguishing inflamed from normal cells[3], we sought to determine if such alterations correlated with each other in biologically specified programs. We also asked how pathogenic immune cell identities in psoriasis lesions, as revealed by scRNA-seq, might evolve during therapeutic treatment of interleukin-23 (IL-23) pathway overactivity.

RESULTS

scRNA-seq of T cells isolated from psoriatic lesions and uninflamed skin reveals both pathogenic Th17/Tc17 activation and suppression of inflammation-suppressive genes

scRNA-seq using the 10x Genomics Chromium droplet-based methodology was performed in tandem with cellular indexing of transcriptomes and epitopes by sequencing (CITE-seq; see STAR Methods) on $CD45^+$ immune cells isolated from lesional skin biopsies of 8 patients with psoriasis, comparable in cohort size to recent scRNA-seq studies (Tables S1 and S2).[4,8] Cutaneous $CD45^+$ cells were also isolated and profiled from seven uninflamed donors. We performed high-resolution unsupervised clustering on $CD3^+$ cells and examined major classes that each represented more than 5% of all T cells in our samples (Table S3). We defined these six T cell classes passing this filter based on their transcriptional and epitope (CITE-seq) markers (Figure 1A), including central memory cells (Tcms), two tissue-resident memory (Trm1 and Trm2) populations, one exhausted and one activated cytotoxic $CD8^+$ T lymphocyte population (CTLex and CTLac, respectively), and regulatory T cells (Tregs). Tcms were $CD45RA^-$(epitope)/*$CD62L^+$*/*$CCR7^+$*, while the two closely related Trm classes were $CD69^+$(epitope)/*$CD103^+$*[9,10] and also *$CXCR6^+$*[11], distinguished from each other by only a small set of non-canonical markers (Table S3). Relative to CTLac, CTLex harbored elevated *PDCD1*, *LAG3*, and *PRF1*. All six classes were

CellPress
OPEN ACCESS

robustly represented in each of our 15 patient samples and in aggregate ranged from 62% to 81% of T cells in each sample (Figure 1B; Table S3; Figure S1).

We next examined differential gene expression in T cell subpopulations in the 8 psoriasis samples against our 7 uninflamed, normal controls using a pseudo-bulk approach.[12,13] Elevation in psoriatic T cells of Th17/Tc17 cytokines such as *IL17A*, *IL17F*, *IL26*,[14] *IFNG*, and *CXCL13*[15] occurred predominantly in a population harboring skin-resident memory T cells markers (Trm1; Figure 1C; Table S4). *IL22* expression was lower and thus less reliable for bulk comparison in these data. A broad array of T cell activation markers such as *CTLA4*, *CD2*, and *CD82* were also elevated in these single cells expressing psoriatic cytokines, as was suppression of a series of inflammation-suppressive transcripts not previously reported as a major feature of T cells in psoriasis or other inflammatory skin diseases. Such transcripts include *ZFP36L2* and *ZFP36*, two tristetraprolin family members previously shown to be capable of repressing inflammatory potential in murine and human T and B cells.[16–19] In fact, among the 140 transcripts repressed >1.5 average log2 fold change (FC) in the Th17/Tc17 cytokine-expressing Trm1 cell class with an adjusted p value <0.01, we also observed multiple other genes known to suppress inflammatory cellular identity, including inhibitors of JAK/STAT (*SOCS1*[20] and *SOCS3*[21]) and Th17 signaling (*CD69*[22]), negative regulators of $CD8^+$ cytotoxic function (*LGALS1*[23]), and promoters of Treg activity (*CCL22*[24]). Downregulation of these inflammation-suppressive genes was also observed in psoriatic samples in other T cell classes, most strongly Tcm and Trm2 (Table S4), although T cells expressing canonical psoriatic cytokines classified primarily in the Trm1 category.

Because Th17 and Tc17 cells have each been reported as arising from skin-resident memory cells,[2,25,26] we were curious if both populations could be identified in our skin-resident memory T cells, which harbored virtually all expression of psoriatic cytokines. Psoriatic T cells in the Trm1 subpopulation were subclassified as $CD4^+$ and $CD8^+$ based on expression of *CD4*, *CD8A*, or *CD8B* (STAR Methods; Table S3) as in other recent studies.[4] As seen in Figure 1D, both the psoriatic $CD4^+$ and $CD8^+$ subpopulations substantively expressed *IL17A*, *IL17F*, *IL26*, and *IFNG*; we hereafter refer to these classes as Th17 and Tc17, respectively.

A *ZFP36L2*-centric cell identity gradient defines pathogenic cytokine and inflammatory transcript expression in psoriatic skin-resident memory T cells on the single-cell level

The detection of these transcriptional differences allowed us to evaluate two differing hypotheses regarding T cell identity in psoriasis. First, upregulation of psoriatic inflammatory transcripts and downregulation of inflammation-suppressive individual gene transcripts might simply occur in an unrelated pattern within Th17/Tc17 single cells. Alternatively, expression of these two gene sets might be coordinately linked in specific, related patterns on the single-cell level. To distinguish these possibilities, we examined correlations between *IL17A* and *IL17F* and other highly expressed transcripts in skin-resident memory T cells. To eliminate artifacts, gene values were batch corrected at the sample level using the CPCA method in the R package iCellR (STAR Methods). Intriguingly, not only was expression of *IL17A* and *IL17F* each highly correlated with one another, and also with *IFNG*, but the greatest levels of anti-correlation were observed against many of the inflammation-suppressive transcripts we described above as downregulated in this class. In fact, by this measure, *ZFP36L2* was the single most anti-correlated transcript out of 16,348 assessed genes versus both *IL17A* ($\delta = -0.58$, $p < 2 \times 10^{-5}$) and *IL17F* ($\delta = -0.65$, $p < 2 \times 10^{-5}$) expression in Th17/Tc17 cells (Figures 2A and S2). A similar, high degree of anti-correlation was also observed between psoriatic cytokine transcripts and *ZFP36*, whose expression closely tracks that of *ZFP36L2* in single T cells (Table S5).

We asked if pro-inflammatory transcripts beyond *IL17A* and *IL17F* were elevated in single memory T cells with the lowest *ZFP36L2* magnitude. Indeed, transcripts demonstrating Spearman anti-correlation against *ZFP36L2* of $\rho < -0.6$ ($p < 2.1 \times 10^{-344}$, 43/16,343 assessed genes) included the psoriasis-related cytokine *IL26*[14] and the T cell activation modulator *CD2*,[27] as well as multiple T cell receptor components (*CD3D* and *CD3G*) and activation markers (*CD81*,[28] *CD82*,[29] and *CD96*[30]) (Figure 2A; Table S5). Other psoriasis-related genes also enriched in the most *ZFP36L2*-deficient cells included the effector chemokine *CXCL13*[15] ($\rho = -0.49$, $p < 5.9 \times 10^{-320}$).

The correlation graphs in Figure 2B show that *ZFP36L2* and inflammatory transcript expression opposed each other in single Th17/Tc17 cells as a continuous gradient rather than, for example, in discrete classes. This gradient was relatively muted in uninflamed Trms and most evident in the transition from Trms in healthy, uninflamed tissue to Th17/Tc17 cells in lesional psoriatic skin. This phenomenon presented visually as the blue, normal T cells observed at the upper left end of each gradient (with high *ZFP36L2* expression and low cytokine expression), in contrast to the dark red lesional T cells extending toward the lower right. Substantial expression of key Th17 cytokines such as *IL17A*, *IL17F*, and *IL26* was restricted sharply to psoriatic single Th17/Tc17 cells whose *ZFP36L2* level was suppressed below that of Trms in normal, uninflamed skin.

It was not clear whether such gradient patterns arose primarily out of either the Th17 or Tc17 subpopulations. We therefore repeated this analysis after compartmentalizing $CD4^+$ and $CD8^+$ subpopulations as described above. As illustrated in Figure S3, the gradients involving *ZFP36L2* were robustly detected in both subpopulations, confirming a recurrent patterning of pathogenic cytokines within both Th17 and Tc17 identities. Thus *ZFP36L2* deficiency, paralleled by depressed *ZFP36* levels, appears to specify a pathogenic inflammatory state in psoriatic T cells, a transcriptional identity largely absent in the corresponding resident-memory T cells isolated from non-inflamed skin (Figure 2B, blue data points).

The prominent anti-correlation between tristetraprolin family members and Th17/Tc17 cytokines also led us to evaluate their relationship beyond human skin T cells. In mice, thymically derived murine $\gamma\delta$ T cells produce IL17 isoforms and drive psoriasiform inflammation. These $\gamma\delta$ T cells express cognate Trm markers, proliferate *in situ* in response to local

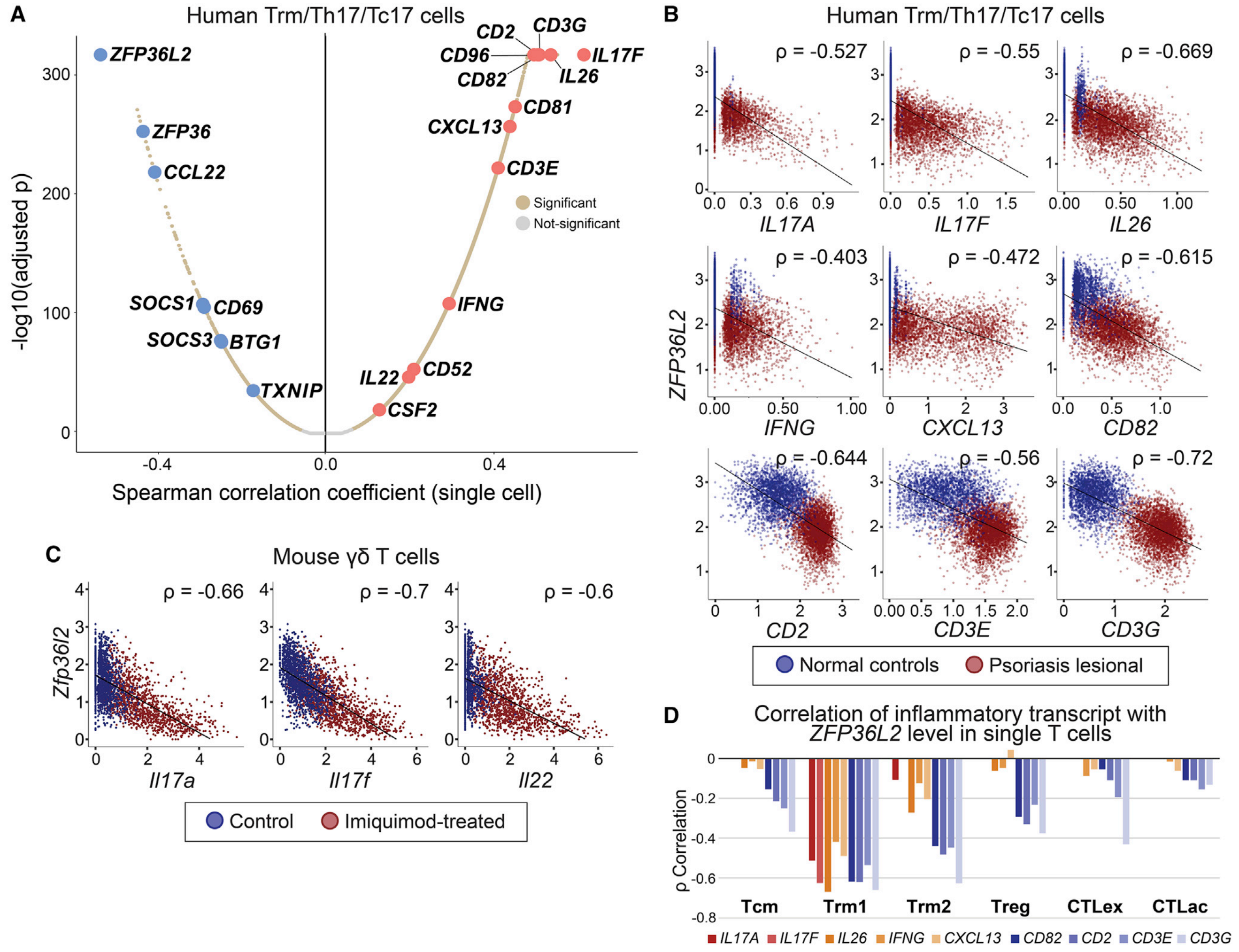

Figure 2. scRNA-seq identifies *ZFP36L2* as the transcript most anti-correlated with psoriatic inflammatory gene expression in skin-resident T cells

(A) Positive correlation of *IL17A* expression in single Trm/Th17/Tc17 cells with expression of pro-inflammatory transcripts and cytokines (right half of graph, labeled in red). Deficiency in *IL17A*-expressing single T cells of inflammation-suppressive genes such as *ZFP36L2*, *ZFP36*, and *BTG1* is shown as negative Spearman correlations (left half of graph, labeled in blue). Data from 8 psoriasis and 7 normal samples. An analogous correlation graph for *IL17F* is shown in Figure S2.

(B) *ZFP36L2* suppression predicts induction of numerous other pro-inflammatory mediator transcripts from Th17/Tc17 in lesional psoriatic skin from this study (red, all 8 samples pooled) versus healthy controls (blue, all 7 samples pooled). The y axis shows *ZFP36L2* expression, the x axis shows imputed cytokine transcript levels, and each point represents a single T cell.

(C) scRNA-seq from prior studies of CD45^{+} cutaneous immune cells isolated from imiquimod-provoked (red, 3 samples pooled) versus control treated mice (blue, 3 samples pooled)[31] show maximal expression of *IL17A*, *IL17F*, and *IL22* with highest *ZFP36L2* suppression. The y axis shows *ZFP36L2* expression, the x axis shows imputed cytokine transcript levels, and each point represents a single γδ T cell. For both axes, the standard imputed expression value (iCellR) has been normalized and log-transformed (Seurat; STAR Methods).

(D) Relationship between *ZFP36L2* loss and inflammatory mediator expression seen in all major skin T cell classes but strongest in skin-resident memory T cells.

tissue cytokines, and do not freely recirculate out of the skin and are thus frequently regarded as skin-resident analogs in psoriasis models. In re-analyzing single-cell data from imiquimod-provoked γδ T cells in mice,[31] we again observed a steep anti-correlation between levels of *Zfp36l2* and the Th17/Tc17 cytokines *Il17a*, *Il17f*, and *Il22* (Figure 2C). In data from stimulated human peripheral T cells,[7] we also found that *IL17F* was expressed almost exclusively in low *ZFP36L2*-expressing single cells (Figure S4), indicating that this relationship extends to psoriatic cytokine expression outside of tissue-resident settings.

While we primarily detected and modeled significant psoriatic cytokine expression in cells falling in the Trm1 subpopulation, anti-correlation of other inflammatory markers against *ZFP36L2/ZFP36* levels was also observed in our Tcm, Treg, and CD8^{+} classes (Figure 2D; Table S5), suggesting that tristetraprolin family member expression pervasively defines inflammatory cell identity across skin T cell classes.

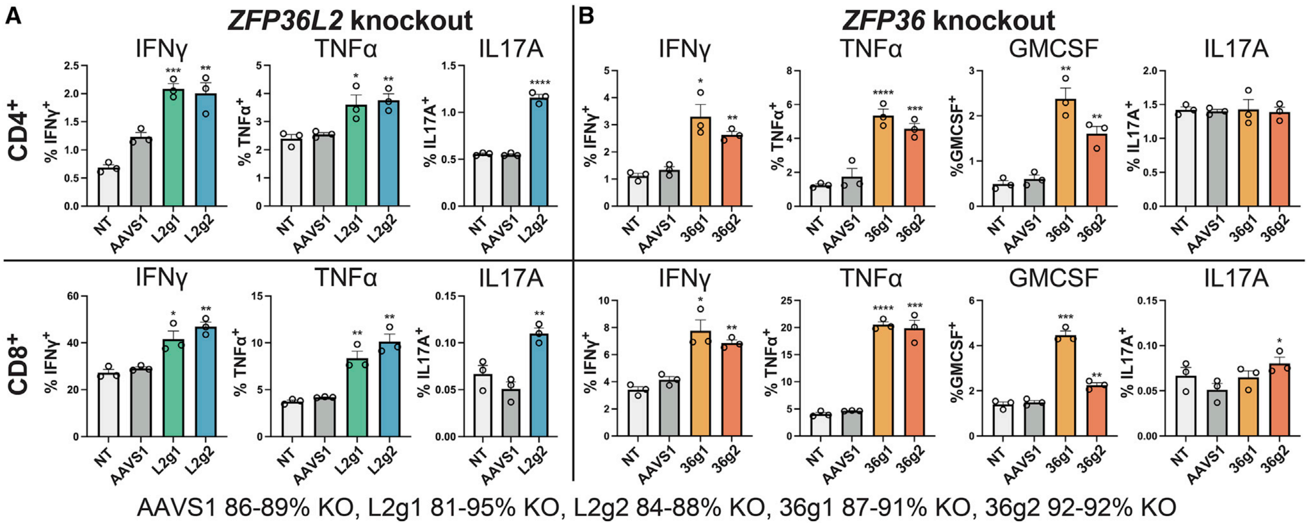

Figure 3. *ZFP36L2* and *ZFP36* knockout increases the intracellular concentration of 3′ ARE-containing cytokines

Frequency of $CD4^+$ (top rows) and $CD8^+$ (bottom rows) T cell staining for the cytokines indicated on the y axis.

(A) Frequency of cytokine-positive cells from *ZFP36L2* knockout T cells shown in green for each of two gRNAs (L2g1 and L2g2, biological triplicate experiments).

(B) Frequency of cytokine-positive cells from *ZFP36* knockout T cells shown in orange for two gRNAs (36g1 and 36g2, biological triplicate experiments).AAVS1 and NT are AAVS1 safe-harbor-targeting and non-targeting negative control gRNAs. *$p < 0.05$, **$p < 0.01$, ***$p < 0.001$, Student's two-sample, two-tailed t tests. Error bars: SEM. Sanger gene-inactivation percentages displayed at bottom. Selected cytokine assessments, including the L2g1 *ZFP36L2* guide for IL-17A, were performed using an additional donor (Figure S5).

ZFP36L2 or *ZFP36* knockout in primary $CD4^+$ and $CD8^+$ T cells elevates protein and transcript expression of 3′ ARE-containing cytokines

We were curious if marked suppression of *ZFP36L2* and *ZFP36* mRNA abundance simply correlated with increased expression of key cytokines or, instead, functionally elevated their levels. Both ZFP36L2 and ZFP36 bind AU-rich elements (AREs) in the 3′ untranslated region (UTR) of mRNA transcripts, directly inhibiting ribosomal access and translation. Tristetraprolin family members have been shown to suppress ARE-harboring cytokine transcript levels in mouse T cell populations[32] and in human Tregs.[33] We utilized CRISPR-Cas9 ribonucleoprotein complexes to genetically disrupt both loci in primary peripheral T cells because of the technical challenges in obtaining human cutaneous T cells in sufficient numbers for these assays. As the relationship between *ZFP36L2* and Th17/Tc17 cytokine production was broadly conserved in both tissue-resident and other skin T cell classes (Figures 2B and 2D), we targeted knockout to bulk $CD4^+$ and $CD8^+$ T cells. Two distinct guide RNAs (gRNAs) were designed for both *ZFP36L2* and *ZFP36*; greater than 81% allele knockout was confirmed for each gRNA, using both Sanger-based (Synthego ICE[34]) and next-generation sequencing approaches (knockout percentages in Table S6; exemplar Sanger traces shown in Figure S5). A negative control gRNA for the human *AAVS1* safe-harbor integration locus was tested to control for non-specific effects of Cas9-mediated double-strand breaks.

Using biological triplicates of our CRISPR-Cas9 ribonucleoprotein-based knockouts of *ZFP36L2* and *ZFP36* (Figure S5), we assessed levels of 3′ ARE-containing cytokines by flow cytometry. Tumor necrosis factor alpha (TNF-α) and granulocyte macrophage-colony-stimulating factor (GM-CSF) are known to be elevated in psoriatic T cells,[35] although usually below detection limits for 10x Genomics 3′ scRNA-seq analysis.[4] As shown in Figure 3A, knockout of ZFP36L2 in both $CD4^+$ and $CD8^+$ T cells significantly induced interferon gamma (IFNγ), TNF-α, and IL-17A. By comparison, *ZFP36* knockout induced IFNγ and TNF-α in both populations but did not appreciably affect IL-17A expression (Figure 3B). GM-CSF was assayed specifically for the ZFP36 knockout, based on a previously reported regulatory relationship.[36]

The observed cytokine induction under these conditions was substantial. *ZFP36L2* knockout resulted in as much as a 1.6-fold increase in IFNγ-positive $CD8^+$ T cells, from 29% to 47% (guide L2g2, $p < 0.01$; Figure 3A, second row). *ZFP36L2* knockout also induced TNF-α 2.4-fold in $CD8^+$ T cells, from 4.2% to 10% positivity (guide L2g2, $p < 0.01$), and IL-17A 2.1-fold ($p < 0.0001$; Figures 3A and S5). Assessments for TNF-α and IFNγ were repeated in CRISPR knockout $CD4^+$ and $CD8^+$ T cells obtained from a different donor for one guide (Figure S5).

As the binding of tristetraprolin family members to cytokine mRNA also targets them for deadenylation and degradation,[37] we also assessed *ZFP36L2* influence on transcript levels. We bulk RNA-seq-profiled $CD4^+$ primary human T cells after *ZFP36L2* disruption performed with three distinct donors (Table S7; STAR Methods). Applying the differential expression analysis package sleuth to these data,[38] among transcripts for secreted cytokines, we detected that *ZFP36L2* knockout significantly increased levels of *IL17A* and *IFNG*, which are elevated in *ZFP36L2*-deficient T cells ($p < 0.01$; Figure 2A). However, other pro-inflammatory genes elevated in *ZFP36L2*-deficient T cells but lacking 3′ AREs, such as *CXCL13* and *CD82*, were not induced by its disruption, suggesting that there are additional

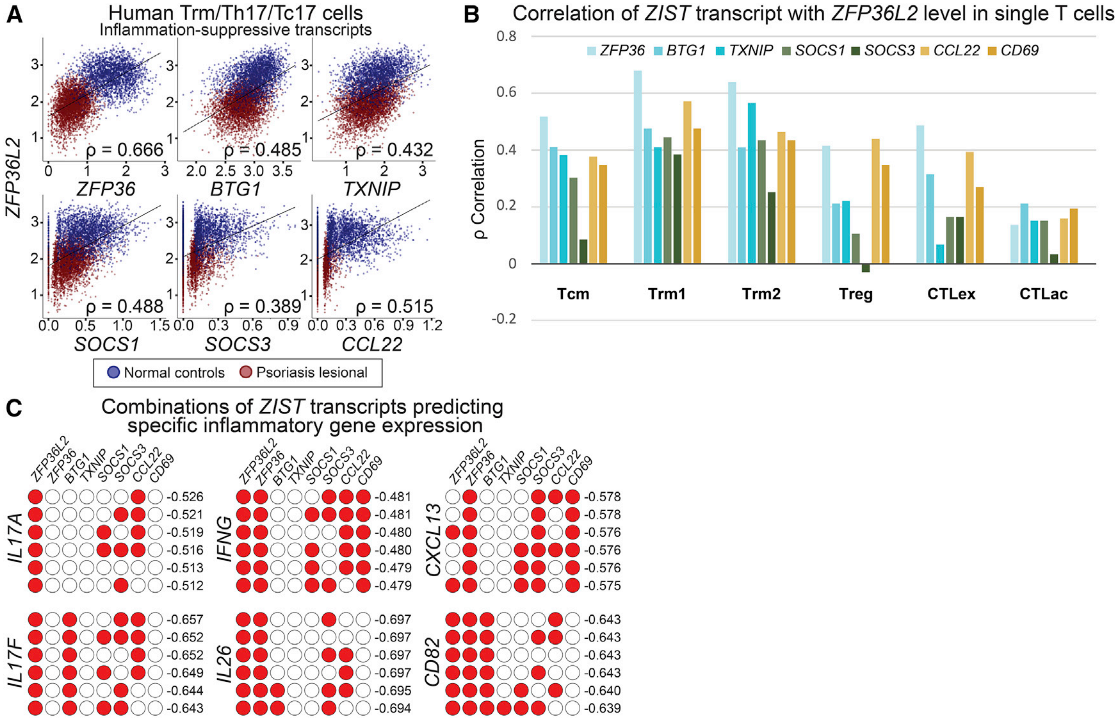

Figure 4. Loss of a coordinated inflammation-suppressive program centered on *ZFP36L2* defines inflamed psoriatic skin-resident memory cells

(A) *ZFP36L2* expression coordinates with that of numerous other global inflammatory suppressors in Th17/Tc17 single cells in lesional psoriatic skin (red, all 8 samples pooled) compared with healthy controls (blue, all 7 samples pooled), a program we term the *ZFP36L2* inflammation suppressive transcript program (*ZIST*). The y axis shows *ZFP36L2* expression, the x axis shows transcript levels for the specified suppressor gene, and each point represents a single T cell.

(B) Correlation between most *ZIST* genes is statistically significant in different T cell subclasses but is strongest in Trm1.

(C) Combinatoric analysis shows top six *ZIST* gene groups whose combined downregulation best predicts individual inflammatory transcript expression (Spearman correlation coefficient displayed at right for gene named at left, different *ZIST* transcript subsets denoted by filled red circles in each row), revealing predictive associations of *ZFP36L2* for *IL17A*, as well as of *BTG1* for *CD82.*

mechanisms responsible for their upregulation. To further distinguish direct targets of tristetraprolin proteins in T cells, we examined data from a recent study employing a binding-based biochemical assay to identify Zfp36 binding partners in wild-type $CD4^+$ cells.[19] Of 382 transcripts showing high-magnitude suppression versus *ZFP36* level in our study ($\rho < -0.5$, $p < 0.01$), 199 were also identified in this screen (Table S5).

ZFP36L2 defines programmatic attenuation of other inflammation-suppressive transcripts in rash lesional T cells

Our findings support a model in which repressed *ZFP36L2* and *ZFP36* expression, in psoriatic Th17/Tc17 cells, elevates the abundance of 3′ ARE-containing cytokines. However, many other pro-inflammatory transcripts that were elevated in *ZFP36L2*/*ZFP36*-deficient T cells lack such 3′ regulatory elements (e.g., *CD2*, *CD82*, and T cell receptor components), suggesting a different regulatory cause. We speculated that other inflammation-suppressive genes might be downregulated in concert with *ZFP36L2* in single Th17/Tc17 cells, more broadly activating an inflammatory state. We thus examined the 50 transcripts most correlated with *ZFP36L2* in Trms/Th17/Tc17 cells ($\rho > 0.4$, <0.2% of assessed transcripts, $p < 6.1 \times 10^{-364}$, excluding ribosomal or mitochondrial transcripts; Table S5) for prior biological evidence of immune pathway restriction. Strikingly, many inflammation-suppressing genes that we noted earlier as downregulated in psoriatic T cells were found in this set, including *ZFP36*, *SOCS1*, *SOCS3*, *CCL22*, and *CD69*, as well as the global quiescence enforcers *BTG1*[39] and *TXNIP*[40] (Figure 4A). We thus termed these genes, which appear coherently suppressed in the highest cytokine-expressing single Th17/Tc17 cells, the *ZFP36L2* inflammation-suppressive transcriptional program, or *ZIST*. *ZFP36L2* correlates with other *ZIST* genes in both skin-resident $CD4^+$ and $CD8^+$ subfractions (Figure S3). However, its statistical correlation is weaker in the Treg subpopulation and $CD8^+$ cells outside of the Trm1 cluster (Figure 4B). *ZIST* transcripts were not identified as significantly altered in T cells in a study of collagenase artifacts in human tissue processing.[41]

We hypothesized that the elevated expression of multiple inflammation-suppressive genes (i.e., *ZIST*) normally restricts T cell inflammatory tone. Exogenous stimulation then downregulates *ZIST* components, an effect observed for both *ZFP36L2* and *ZFP36* in peripheral T cells,[7] potentiating inflammation.

While expression of *ZIST* members appears strongly correlated within Trms/Th17/Tc17 cells, we were curious if their individualistic effects on specific inflammatory targets might still be distinguishable on a single-cell level. Such associations would implicate individual *ZIST* members as regulators of distinct aspects of psoriatic T cell activation. To test this hypothesis, we implemented a single-cell, combinatoric approach to test correlations between (1) transcript levels of all possible, non-repeating sets of predictor (i.e., *ZIST*) genes and (2) expression of individual inflammatory mediator genes described above.

By testing all possible *ZIST* sets in their prediction of inflammatory transcript expression, this approach is capable of testing correlative effects of groups of genes but also quantitatively discerning if components of sets have outsize effects on targets. Briefly, the batch-corrected matrices described above were passed to Monocle3, and all possible combinations of predictor (*ZIST*) genes were then summed using the aggregate_gene function, in order to calculate Spearman rank correlation with respect to inflammatory mediator target genes (Table S8; STAR Methods).

These analyses reveal two intriguing features of psoriatic inflammatory activation in resident T cell populations. First, while downregulated *ZFP36L2* and *ZFP36* rank as the best individual predictors of inflammatory gene expression, combinations of suppressed *ZIST* program genes consistently perform even more strongly (Figure 4C; Table S5). Therefore, we hypothesize that a yet unidentified global regulator coordinately suppresses these *ZIST* program genes, producing a single, major inflammatory gradient in T cells from psoriatic skin. Secondly, even within this dominant axis, our combinatorial analysis can still distinguish gene-specific patterns. For example, *ZFP36L2* suppression is more predictive than that of *ZFP36* for *IL17A* ($\rho = -0.513$ versus -0.416) and *IL17F* ($\rho = -0.625$ versus -0.486) expression. In contrast, combined *ZFP36L2* and *ZFP36* suppression better predict expression of *IFNG* ($\rho = -0.481$ versus -0.418) and *CXCL13* (-0.578 versus -0.490) than *ZFP36L2* alone.

Notably, these findings reflect our CRISPR-based experimental data that ZFP36L2, but not ZFP36, represses IL-17A in human $CD4^+$ and $CD8^+$ T cells, while both suppress IFNγ (Figure 3). Figure 4D also shows an influence of *BTG1* suppression on *CD82* expression not observed for cytokine targets; this prediction agrees with the experimental induction of *CD82* by *BTG1* knockout,[39] suggesting that our model may predict specific regulatory relationships for additional validation. While these analyses are intended for examination of broad trends rather than ascribing significance to each individual *ZIST* combination, we also implemented a stepwise model comparison algorithm using R/cocor, establishing significance between combinatoric pairs (STAR Methods; Table S8).

ZIST program expression defines a trajectory reflecting an inherent molecular gradient from normal to psoriatic skin-resident memory cells

Given the coherence of the *ZIST* program in Th17/Tc17 single cells, we thought that its magnitude might reveal an inherent trajectory defining molecular evolution from normal to inflamed T cells in psoriasis. To examine such a natural trajectory, also referred to as a pseudotime, we optimized a manifold consisting of Trms/Th17/Tc17 cells from all 15 samples and overlaid expression of key transcript sets. Briefly, we calculated an integrated expression value for each gene program within each cell, representing the summed standardized values of each constituent gene within a set. The cells were then organized in two-dimensional space based on a rooted trajectory.

After batch correction, this trajectory shows evenly distributed contributions from all our samples (Figure 5A), and unsurprisingly, its rooted, unsupervised gradient is defined by a transition from normal Trms to psoriatic Th17/Tc17 cells (Figure 5B). This trajectory is not strongly biased by either *CD4*/*CD8* expression (Figure 5C), nor is it a product of central memory markers such as *CCR7* or *SELL* (Figure 5D). As we speculated, the major transition from normal to pathologic T cells is strongly anti-correlated with *ZIST* program magnitude (Figure 5E) and is correlated with cytokine and inflammatory mediator expression (Figures 5F and 5G), a phenomenon that is robustly represented on the individual-sample level (Figure S6). The rooted trajectory is notable for its linear nature, with few outlying developmental sinks (Figure 5H). This analysis thus reinforces a model in which psoriatic skin-resident T cells harbor an inflammatory identity inversely related to expression of *ZIST* program members.

Therapeutic IL-23 blockade drastically reduces Th17/Tc17 cell frequency, but residual Trms in healed psoriasis lesions show persistent *ZIST* program suppression

We next sought to understand how treatment of psoriasis using IL-23 blockade would affect our inflammation-suppressive gradient. Although *IL17A* and *IL17F* cytokine expression should be largely quenched by IL-23 blockade, the pro-inflammatory identity represented by *ZIST* suppression might persist in residual T cells. To assess this hypothesis, we isolated and performed scRNA-seq on lesional T cells from three patients with psoriasis prior to initiation on the IL-23-blocker tildrakizumab. After at least 8 weeks of biologic treatment, which produced a 75% or greater reduction in psoriasis area and severity index score in all three patients, we re-biopsied and profiled clinically resolved skin adjacent to the original sample sites for comparison. Tildrakizumab dramatically reduces Th17/Tc17 cells in psoriatic lesions, an expected feature of blocking IL-23 signaling to skin-resident memory cells, both in absolute number (Figure 6A) and, importantly, as a relative percentage of T cell class (Table S9).

However, in residual resident memory T cells of the Trm1 class isolated from clinically resolved psoriasis lesions, our scRNA-seq analysis also shows persistent abnormal suppression of *ZIST* elements, with mid-treatment versus normal comparison Kruskal-Wallis p values $<2 \times 10^{-16}$ for all transcripts (Figure 6B; patient-level data in Table S9). Plotting *ZIST* program member expression against *ZFP36L2* levels in Trms/Th17/Tc17 cells from these pre- and mid-treatment psoriasis lesions shows that despite resolution of visible inflammatory plaques, the overall inflammatory-suppressive gradient in T cells remains sharply suppressed (Figure 6C). Some, but not all, inflammatory transcripts lacking AREs also appear persistently elevated in the context of clinical improvement, including *CD2* and *CD3G* (Table S9). These data thus reveal that skin-resident memory cells under IL-23 blockade continue to harbor a molecularly primed state closely resembling that in inflamed skin. Such

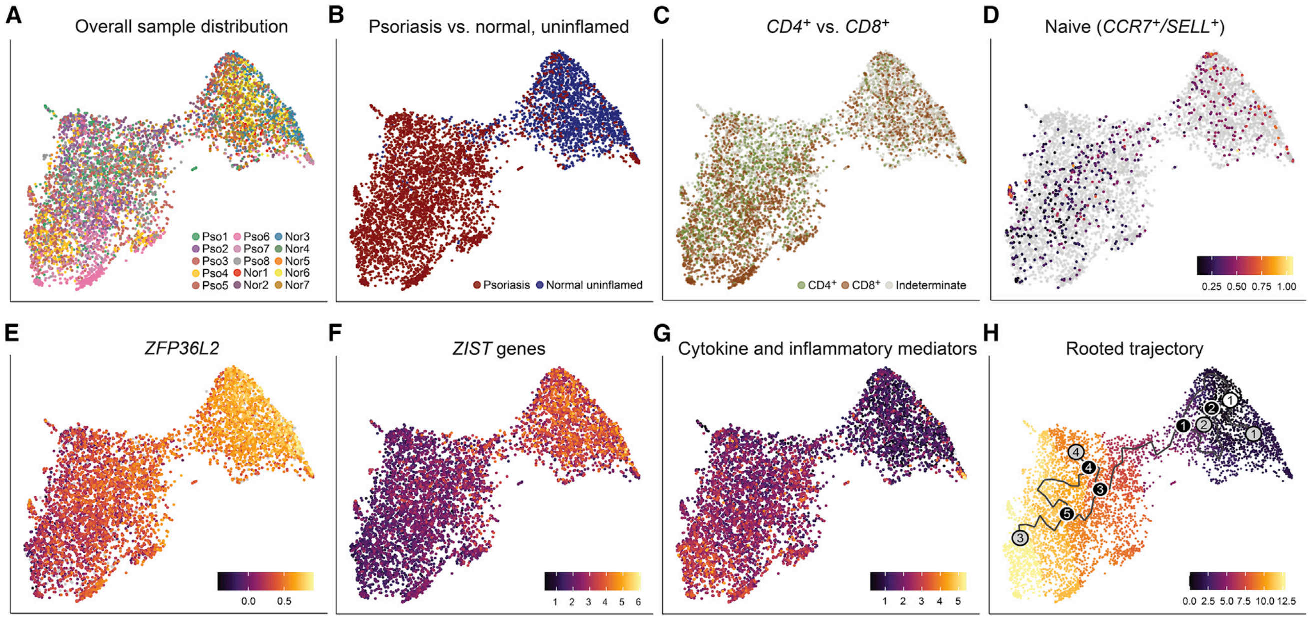

Figure 5. The *ZIST* program gradient mirrors an inherent molecular trajectory organizing the transition from uninflamed to psoriatic skin-resident memory T cells

(A) A pseudotime constructed from a batch-corrected manifold shows distributed representation of all samples.

(B–D) Uninflamed Trms and psoriatic Th17/Tc17 cells are clearly distinguished in this pseudotime, distinct from (C) *CD4/CD8* or (D) central memory marker expression (e.g., *CCR7/SELL*).

(E–G) *ZFP36L2* and *ZIST* expression reflects the natural pseudotime, in inverse relation to (G) a mapping of inflammatory genes *IL17A*, *IL17F*, *IL26*, *IFNG*, *CXCL13*, *CD2*, *CD82*, and *CD3E/G*.

(H) A rooted trajectory shows a single dominant path from uninflamed cells (root node, white circle 1), with linearly arrayed transitional states (black circles) and divergent development sinks/end states (gray circles).

pro-inflammatory abnormalities in cell identity nominate a mechanism by which psoriatic lesions may rapidly recur after withdrawal of treatment.

DISCUSSION

Th17/Tc17 cytokines are well-established to play a central, therapeutically actionable role in the development of psoriasis, but these pathways only partly explain the clinical course of skin inflammatory disease. Dynamic, secondary mechanisms likely activate immune cell populations during cutaneous flares, which are rapid and unpredictable. Our analysis of single-cell sequencing data from psoriasis lesions reveals a reactive layer of heterogeneity embedded in cutaneous T cells. Specifically, a transcriptional single-cell identity involving multiple inflammation-suppressive regulators (described here as the *ZIST* program) appears to play a dominant role in defining the most pathogenically activated psoriatic T cells. *ZIST* program members like *ZFP36L2* and *ZFP36* post-transcriptionally suppress pre-formed Th17/Tc17 cytokine transcripts and are known to be rapidly de-activated,[42,43] nominating a mechanism by which chronic rashes might rapidly flare.

Some *ZIST* members outside of *ZFP36L2* and *ZFP36* are well-characterized global regulators of T cell activation, such as *BTG1*, or SOCS proteins, which appear to substantially inhibit *JAK/STAT* signaling.[44] Others, such as *CCL22*, may lower T cell inflammatory tone both by direct action on Tregs[24] and also dendritic cell populations.[45] Like the numerous genes that are upregulated in and augment the inflammatory capacity of inflamed immune cells, the exact delineation of such a program is partially arbitrary and likely to be substantially tissue specific.

Our findings reveal that *ZIST* inflammation-suppressive transcripts exist in a single-cell continuum. This gradient is then coordinately shifted downward in psoriatic lesions, relieving suppression of inflammation and driving cytokine expression in the most *ZIST*-depressed subpopulation. In fact, we find that *IL17F* and *IL26* expression emerges only in those psoriatic T cells whose *ZIST* expression is depressed below the range observed in normal control samples (Figure 2B), underscoring the tight link between this gradient and pathogenic inflammation. We investigate this regulatory pattern primarily in our Trm1 subpopulation, in which Th17/Tc17 cytokines are expressed in psoriasis samples and can be studied directly, but other inflammatory markers negatively correlate with *ZFP36L2*-deficiency in other T cell classes (Figure 2D). A recent study also observed *ZFP36L2* suppression in stimulated Tregs, as seen in our data, apparently elevating Treg tone and perhaps representing a feedback mechanism that is overwhelmed in the context of pathogenic inflammation.[33]

We propose that in psoriatic lesion development, general, exogenous inflammatory insults suppress elements of the *ZIST* program in healthy Trms, leading to enhanced pathogenic psoriatic cytokine and inflammatory mediator transcript levels. An

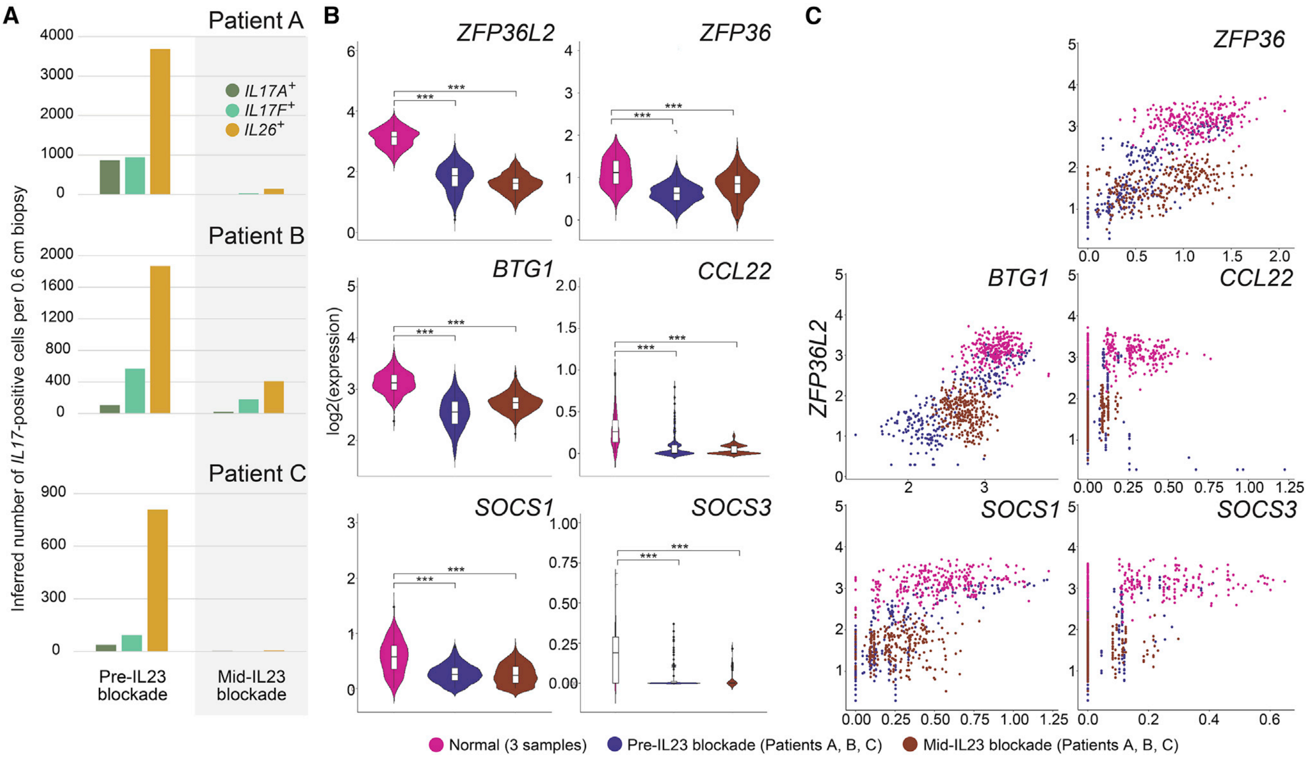

Figure 6. IL-23 blockade with tildrakizumab eliminates most Th17/Tc17 T cells in three treated patients, but residual Trms show persistent suppression of the *ZIST* program

(A) Inferred *IL17A*$^+$, *IL17F*$^+$, and *IL26*$^+$ cell numbers per 6 mm biopsy shown for normal, psoriatic, and mid-IL-23 blockade (>8 weeks) for three individual patients (see Table S6 for CD4$^+$/CD8$^+$ ratios).

(B) *ZIST* member suppression persists in residual, IL-23-blockaded skin-resident T cells (brown) versus normal, uninflamed Trms (mauve), similar to untreated psoriatic Th17/Tc17 cells (blue) despite nearly complete clinical resolution. Three asterisks (***) denote differences in displayed violin plots of $p < 2 \times 10^{-16}$ (Kruskal-Wallis test).

(C) Scatterplots illustrate *ZIST* program members are co-suppressed with *ZFP36L2* in Trm1 class single cells isolated from mid-treatment biopsies, showing persistence of the gradient despite the context of IL-23 blockade.

increasing inflammatory milieu then further depresses *ZIST* levels, as previously shown with *in vitro* suppression of *ZFP36L2* and *ZFP36* in peripheral T cells by T cell receptor (TCR) stimulation.[7,33] Our pre- and mid-tildrakizumab treatment data support this model, in which IL-23 signaling and *ZIST* regulation represent dual, distinguishable inputs, as IL-23 blockade sharply reduces Th17/Tc17 cell density but does not normalize *ZIST* suppression in Trms from healed psoriatic lesions. Although our patients represent a limited initial cohort that must be expanded, such a molecularly primed, inflammatory cell identity persisting despite IL-23 blockade might accelerate recurrence of psoriatic lesions after withdrawal of treatment.

ZFP36L2 and *ZFP36* attenuation help explain cytokine induction in *ZIST*-deficient T cells but are unlikely to directly elevate the numerous other pro-inflammatory transcripts lacking 3′ ARE sequences, their binding target. Such inflammatory markers include *CD2*, *CD3G*, and *CD82*, which are known to establish the T cell immunological synapse and maintain signaling downstream of the TCR,[46] and their upregulation is likely to further activate more greatly *ZIST*-deficient single T cells. Potential sources for regulation of these other inflammatory markers include *ZIST* members such as *BTG1*, whose suppression tracks specifically with *CD82* elevation (Figure 4D) and which is not affected by *ZFP36L2/ZFP36* knockdown (Table S7), a relationship that has been mechanistically validated in murine T cells.[39] Therefore, the combinatoric model we present here may be used to predict and further experimentally validate other suppressor-inflammatory mediator relationships.

The persistence of *ZIST* gradient abnormalities in psoriasis despite IL-23 blockade nominates this regulatory mechanism as an intriguing, complementary therapeutic target in overactivity of the Th17/Tc17 axis, as does recent work indicating that fibroblasts may also suppress psoriatic inflammation through ZFP36L2.[47] It also remains to be understood whether this transcriptional gradient acts similarly on Th2 cytokines in atopic dermatitis or in other chronic skin inflammatory disorders. One key technical limitation of our study is the inability to enhance activity of *ZFP36L2* or other *ZIST* elements in human skin. The tightly linked expression of *ZIST* program elements strongly suggests that a yet undiscovered global regulator produces this gradient cell identity, analogous to factors such as FOXO1 and KLF2 that guide the transition from naive to memory cells. A means to selectively activate ZFP36L2 and other *ZIST* genes in human T cells remains an important milestone for future work.

Limitations of the study

This study is limited by the relatively small number of samples analyzed, given the current costs and technical challenges of scRNA-seq. It is also not possible to formally exclude influences of tissue dissociation on the skin-resident T cell transcriptional profiles we report here. Finally, we lack the technical capability to directly genetically modify T cells isolated from human skin, limiting direct, *in vitro* validation of the inflammation suppression function of our candidates. These technical bounds in genetic manipulation also restrict our capacity to measure the aggregate impact of downregulation of multiple inflammation-suppressive transcripts in our T cell gradients.

STAR★METHODS

Detailed methods are provided in the online version of this paper and include the following:

- KEY RESOURCES TABLE
- RESOURCE AVAILABILITY
  - Lead contact
  - Materials availability
  - Data and code availability
- EXPERIMENTAL MODEL AND SUBJECT DETAILS
  - Patient characteristics
- METHOD DETAILS
  - Skin sample collection, single cell RNA-seq, and CITE-seq processing
  - Single cell RNA sequencing data processing
  - Dimensional reduction and unsupervised clustering
  - scRNA-seq correlation analysis
  - Trm1 manifold and developmental trajectory inference
  - Combinatorial single-cell inflammatory mediator prediction analysis
  - Preparation of CRISPR/CAS9 Ribonucleoprotein particles
  - Donor T cell nucleofection
  - PCR-based validation of CRISPR-based T cell gene knockout
  - Intracellular cytokine assays of CRISPR-modified T cells
  - mRNA isolation and bulk RNA-seq of CRISPR-modified CD4$^+$ T cells
  - Bulk RNA-seq analysis
- QUANTIFICATION AND STATISTICAL ANALYSIS
  - Statistical analysis for flow cytometry data
  - Statistical analysis for scRNA-seq and bulk RNA-seq data

SUPPLEMENTAL INFORMATION

Supplemental information can be found online at https://doi.org/10.1016/j.xcrm.2022.100715.

ACKNOWLEDGMENTS

We wish to thank Rachel Sevey for help with figure illustration and Bahram Razani, MD, PhD, for a critical reading of the manuscript. R.J.C. and J.B.C. are supported by a LEO Foundation grant, Sun Pharmaceuticals, and Sanofi. J.B.C. is supported by the National Institute of Arthritis and Musculoskeletal and Skin Diseases of the National Institutes of Health K08AR067243 and the National Psoriasis Foundation. A.M. holds a Lloyd J. Old STAR award from the Cancer Research Institute, was an investigator at the Chan Zuckerberg Biohub, held a Career Award for Medical Scientists from the Burroughs Wellcome Fund, and has received funds from Parker Institute for Cancer Immunotherapy and the Innovative Genomics Institute. The Marson lab has received research support from Juno Therapeutics, Epinomics, Sanofi, GlaxoSmithKline, Gilead, and Anthem.

AUTHOR CONTRIBUTIONS

C.P.C., Y.L., J.B.C., and R.J.C. designed the study. E.K., J.B.C., and R.J.C. supervised sample collection and processing. C.P.C., Y.L., P.H., and R.S. performed sample preparation and analysis. C.P.C., Y.L., M.T., A.S., E.P., H.W., Y.S., J.C., and S.B.V.R. performed computational analysis. J.P.N. performed histopathology. C.P.C., Y.L., J.B.C., and R.J.C. wrote the manuscript with contributions from R.S., A.M., M.T., E.P., H.W., A.S., E.K., J.P.N., P.H., S.W.K., S.C.B., and S.B.V.R.

DECLARATION OF INTERESTS

A.M. is a cofounder of Arsenal Biosciences, Spotlight Therapeutics, and Survey Genomics. A.M. serves on the boards of directors at Spotlight Therapeutics and Survey Genomics and is board observer (and former member of the board of directors) at Arsenal Biosciences. A.M. is a member of the scientific advisory boards of Arsenal Biosciences, Spotlight Therapeutics, Survey Genomics, and NewLimit. A.M. owns stock in Arsenal Biosciences, Spotlight Therapeutics, NewLimit, Survey Genomics, PACT Pharma, and Merck. A.M. has received fees from Arsenal Biosciences, Spotlight Therapeutics, NewLimit, 23andMe, PACT Pharma, Juno Therapeutics, Trizell, Vertex, Merck, Amgen, Genentech, AlphaSights, Rupert Case Management, Bernstein, and ALDA. A.M. is an investor in and informal advisor to Offline Ventures and a client of EPIQ.

INCLUSION AND DIVERSITY

We worked to ensure gender balance in the recruitment of human subjects. We worked to ensure ethnic or other types of diversity in the recruitment of human subjects. One or more of the authors of this paper self-identifies as an underrepresented ethnic minority in science. One or more of the authors of this paper self-identifies as a member of the LGBTQ+ community. One or more of the authors of this paper received support from a program designed to increase minority representation in science. While citing references scientifically relevant for this work, we also actively worked to promote gender balance in our reference list. The author list of this paper includes contributors from the location where the research was conducted who participated in the data collection, design, analysis, and/or interpretation of the work.

Received: January 4, 2022
Revised: May 21, 2022
Accepted: July 14, 2022
Published: August 16, 2022

REFERENCES

1. Lowes, M.A., Kikuchi, T., Fuentes-Duculan, J., Cardinale, I., Zaba, L.C., Haider, A.S., Bowman, E.P., and Krueger, J.G. (2008). Psoriasis vulgaris lesions contain discrete populations of Th1 and Th17 T cells. J. Invest. Dermatol. *128*, 1207–1211.
2. Cheuk, S., Wikén, M., Blomqvist, L., Nylén, S., Talme, T., Ståhle, M., and Eidsmo, L. (2014). Epidermal Th22 and Tc17 cells Form a Localized disease memory in clinically healed psoriasis. J. Immunol. *192*, 3111–3120.
3. Liu, Y., Wang, H., Taylor, M., Cook, C., Martínez-Berdeja, A., North, J.P., Harirchian, P., Hailer, A.A., Zhao, Z., Ghadially, R., et al. (2022).

Classification of human chronic inflammatory skin disease based on single-cell immune profiling. Sci. Immunol. *7*, eabl9165.

4. Reynolds, G., Vegh, P., Fletcher, J., Poyner, E.F.M., Stephenson, E., Goh, I., Botting, R.A., Huang, N., Olabi, B., Dubois, A., et al. (2021). Developmental cell programs are co-opted in inflammatory skin disease. Science *371*, eaba6500.
5. Hughes, T.K., Wadsworth, M.H., Gierahn, T.M., Do, T., Weiss, D., Andrade, P.R., Ma, F., de Andrade Silva, B.J., Shao, S., Tsoi, L.C., et al. (2020). Second-strand synthesis-based Massively Parallel scRNA-seq reveals cellular states and molecular features of human inflammatory skin Pathologies. Immunity *53*, 878–894.e7.
6. Cheng, J.B., Sedgewick, A.J., Finnegan, A.I., Harirchian, P., Lee, J., Kwon, S., Fassett, M.S., Golovato, J., Gray, M., Ghadially, R., et al. (2018). Transcriptional Programming of normal and inflamed human Epidermis at single-cell resolution. Cell Rep. *25*, 871–883.
7. Cano-Gamez, E., Soskic, B., Roumeliotis, T.I., So, E., Smyth, D.J., Baldrighi, M., Willé, D., Nakic, N., Esparza-Gordillo, J., Larminie, C.G.C., et al. (2020). Single-cell transcriptomics identifies an effectorness gradient shaping the response of CD4 + T cells to cytokines. Nat. Commun. *11*, 1801.
8. Bangert, C., Rindler, K., Krausgruber, T., Alkon, N., Thaler, F.M., Kurz, H., Ayub, T., Demirtas, D., Fortelny, N., Vorstandlechner, V., et al. (2021). Persistence of mature dendritic cells, TH2A, and Tc2 cells characterize clinically resolved atopic dermatitis under IL-4Rα blockade. Sci. Immunol. *6*, eabe2749.
9. Clark, R.A. (2015). Resident memory T cells in human health and disease. Sci. Transl. Med. *7*, 269rv1.
10. Willemsen, M., Linkutė, R., Luiten, R.M., and Matos, T.R. (2019). Skin-resident memory T cells as a potential new therapeutic target in vitiligo and melanoma. Pigment Cell Melanoma Res. *32*, 612–622.
11. Wein, A.N., McMaster, S.R., Takamura, S., Dunbar, P.R., Cartwright, E.K., Hayward, S.L., McManus, D.T., Shimaoka, T., Ueha, S., Tsukui, T., et al. (2019). CXCR6 regulates localization of tissue-resident memory CD8 T cells to the airways. J. Exp. Med. *216*, 2748–2762.
12. Crowell, H.L., Soneson, C., Germain, P.-L., Calini, D., Collin, L., Raposo, C., Malhotra, D., and Robinson, M.D. (2020). Muscat detects subpopulation-specific state transitions from multi-sample multi-condition single-cell transcriptomics data. Nat. Commun. *11*, 6077.
13. Robinson, M.D., McCarthy, D.J., and Smyth, G.K. (2010). edgeR: a Bioconductor package for differential expression analysis of digital gene expression data. Bioinformatics *26*, 139–140.
14. Itoh, T., Hatano, R., Komiya, E., Otsuka, H., Narita, Y., Aune, T.M., Dang, N.H., Matsuoka, S., Naito, H., Tominaga, M., et al. (2019). Biological effects of IL-26 on T cell-mediated skin inflammation, including psoriasis. J. Invest. Dermatol. *139*, 878–889.
15. Liu, J., Chang, H.-W., Huang, Z.-M., Nakamura, M., Sekhon, S., Ahn, R., Munoz-Sandoval, P., Bhattarai, S., Beck, K.M., Sanchez, I.M., et al. (2021). Single-cell RNA sequencing of psoriatic skin identifies pathogenic Tc17 cell subsets and reveals distinctions between CD8+ T cells in autoimmunity and cancer. J. Allergy Clin. Immunol. *147*, 2370–2380.
16. Andrianne, M., Assabban, A., La, C., Mogilenko, D., Salle, D.S., Fleury, S., Doumont, G., Van Simaeys, G., Nedospasov, S.A., Blackshear, P.J., et al. (2017). Tristetraprolin expression by keratinocytes controls local and systemic inflammation. JCI Insight *2*, 92979.
17. Galloway, A., Saveliev, A., Łukasiak, S., Hodson, D.J., Bolland, D., Balmanno, K., Ahlfors, H., Monzón-Casanova, E., Mannurita, S.C., Bell, L.S., et al. (2016). RNA-binding proteins ZFP36L1 and ZFP36L2 promote cell quiescence. Science *352*, 453–459.
18. Lee, H.H., Yoon, N.A., Vo, M.-T., Kim, C.W., Woo, J.M., Cha, H.J., Cho, Y.W., Lee, B.J., Cho, W.J., and Park, J.W. (2012). Tristetraprolin down-regulates IL-17 through mRNA destabilization. FEBS Lett. *586*, 41–46.
19. Moore, M.J., Blachere, N.E., Fak, J.J., Park, C.Y., Sawicka, K., Parveen, S., Zucker-Scharff, I., Moltedo, B., Rudensky, A.Y., and Darnell, R.B. (2018). ZFP36 RNA-binding proteins restrain T cell activation and anti-viral immunity. Elife *7*, e33057.
20. Palmer, D.C., and Restifo, N.P. (2009). Suppressors of Cytokine Signaling (SOCS) in T cell differentiation, maturation, and function. Trends Immunol. *30*, 592–602.
21. Carow, B., and Rottenberg, M.E. (2014). SOCS3, a major regulator of Infection and inflammation. Front. Immunol. *5*, 58.
22. Martín, P., Gómez, M., Lamana, A., Cruz-Adalia, A., Ramírez-Huesca, M., Ursa, M.Á., Yáñez-Mo, M., and Sánchez-Madrid, F. (2010). CD69 association with Jak3/Stat5 proteins regulates Th17 cell differentiation. Mol. Cell Biol. *30*, 4877–4889.
23. Cagnoni, A.J., Giribaldi, M.L., Blidner, A.G., Cutine, A.M., Gatto, S.G., Morales, R.M., Salatino, M., Abba, M.C., Croci, D.O., Mariño, K.V., and Rabinovich, G.A. (2021). Galectin-1 fosters an immunosuppressive microenvironment in colorectal cancer by reprogramming CD8+ regulatory T cells. Proc. Natl. Acad. Sci. USA *118*. e2102950118.
24. Rapp, M., Wintergerst, M.W.M., Kunz, W.G., Vetter, V.K., Knott, M.M.L., Lisowski, D., Haubner, S., Moder, S., Thaler, R., Eiber, S., et al. (2019). CCL22 controls immunity by promoting regulatory T cell communication with dendritic cells in lymph nodes. J. Exp. Med. *216*, 1170–1181.
25. Amezcua Vesely, M.C., Pallis, P., Bielecki, P., Low, J.S., Zhao, J., Harman, C.C.D., Kroehling, L., Jackson, R., Bailis, W., Licona-Limón, P., et al. (2019). Effector TH17 cells give Rise to Long-Lived TRM cells that are Essential for an immediate response against Bacterial Infection. Cell *178*, 1176–1188.e15.
26. Whitley, S.K., Li, M., Tabib, T., Weaver, C.T., McGeachy, M.J., Lafyatis, R.A., and Kaplan, D.H. (2021). IL-23 maintains tissue resident memory Th17 cells in murine and psoriatic skin. J. Immunol. *206*, 98.50.
27. Green, J.M., Karpitskiy, V., Kimzey, S.L., and Shaw, A.S. (2000). Coordinate regulation of T cell activation by CD2 and CD28. J. Immunol. *164*, 3591–3595.
28. Witherden, D.A., Boismenu, R., and Havran, W.L. (2000). CD81 and CD28 costimulate T cells through distinct pathways. J. Immunol. *165*, 1902–1909.
29. Shibagaki, N., Hanada, K. i., Yamashita, H., Shimada, S., and Hamada, H. (1999). Overexpression of CD82 on human T cells enhances LFA-1/ICAM-1-mediated cell-cell adhesion: functional association between CD82 and LFA-1 in T cell activation. Eur. J. Immunol. *29*, 4081–4091.
30. Chiang, E.Y., de Almeida, P.E., de Almeida Nagata, D.E., Bowles, K.H., Du, X., Chitre, A.S., Banta, K.L., Kwon, Y., McKenzie, B., Mittman, S., et al. (2020). CD96 functions as a co-stimulatory receptor to enhance CD8+ T cell activation and effector responses. Eur. J. Immunol. *50*, 891–902.
31. Liu, Y., Cook, C., Sedgewick, A.J., Zhang, S., Fassett, M.S., Ricardo-Gonzalez, R.R., Harirchian, P., Kashem, S.W., Hanakawa, S., Leistico, J.R., et al. (2020). Single-cell profiling reveals divergent, globally patterned immune responses in murine skin inflammation. iScience *23*, 101582.
32. Ogilvie, R.L., Sternjohn, J.R., Rattenbacher, B., Vlasova, I.A., Williams, D.A., Hau, H.H., Blackshear, P.J., and Bohjanen, P.R. (2009). Tristetraprolin mediates interferon-gamma mRNA decay. J. Biol. Chem. *284*, 11216–11223.
33. Makita, S., Takatori, H., Iwata, A., Tanaka, S., Furuta, S., Ikeda, K., Suto, A., Suzuki, K., Ramos, S.B.V., and Nakajima, H. (2020). RNA-binding protein ZFP36L2 downregulates Helios expression and suppresses the function of regulatory T cells. Front. Immunol. *11*, 1291.
34. Hsiau, T., Conant, D., Rossi, N., Maures, T., Waite, K., Yang, J., Joshi, S., Kelso, R., Holden, K., Enzmann, B.L., et al. (2019). Inference of CRISPR Edits from Sanger Trace Data.
35. Deng, Y., Chang, C., and Lu, Q. (2016). The inflammatory response in psoriasis: a comprehensive Review. Clin. Rev. Allergy Immunol. *50*, 377–389.
36. Carballo, E., Lai, W.S., and Blackshear, P.J. (2000). Evidence that tristetraprolin is a physiological regulator of granulocyte-macrophage colony-stimulating factor messenger RNA deadenylation and stability. Blood *95*, 1891–1899.

37. Rappl, P., Brüne, B., and Schmid, T. (2021). Role of tristetraprolin in the resolution of inflammation. Biology *10*, 66.
38. Yi, L., Pimentel, H., Bray, N.L., and Pachter, L. (2018). Gene-level differential analysis at transcript-level resolution. Genome Biol. *19*, 53.
39. Hwang, S.S., Lim, J., Yu, Z., Kong, P., Sefik, E., Xu, H., Harman, C.C.D., Kim, L.K., Lee, G.R., Li, H.-B., and Flavell, R.A. (2020). mRNA destabilization by BTG1 and BTG2 maintains T cell quiescence. Science *367*, 1255–1260.
40. Muri, J., Thut, H., and Kopf, M. (2021). The thioredoxin-1 inhibitor Txnip restrains effector T-cell and germinal center B-cell expansion. Eur. J. Immunol. *51*, 115–124.
41. O'Flanagan, C.H., Campbell, K.R., Zhang, A.W., Kabeer, F., Lim, J.L.P., Biele, J., Eirew, P., Lai, D., McPherson, A., Kong, E., et al. (2019). Dissociation of solid tumor tissues with cold active protease for single-cell RNA-seq minimizes conserved collagenase-associated stress responses. Genome Biol. *20*, 210.
42. Noguchi, A., Adachi, S., Yokota, N., Hatta, T., Natsume, T., and Kawahara, H. (2018). ZFP36L2 is a cell cycle-regulated CCCH protein necessary for DNA lesion-induced S-phase arrest. Biol. Open *7*, bio031575.
43. Brooks, S.A., Connolly, J.E., and Rigby, W.F.C. (2004). The role of mRNA turnover in the regulation of tristetraprolin expression: evidence for an extracellular signal-regulated kinase-specific, AU-rich element-dependent, autoregulatory pathway. J. Immunol. *172*, 7263–7271.
44. Liau, N.P.D., Laktyushin, A., Lucet, I.S., Murphy, J.M., Yao, S., Whitlock, E., Callaghan, K., Nicola, N.A., Kershaw, N.J., and Babon, J.J. (2018). The molecular basis of JAK/STAT inhibition by SOCS1. Nat. Commun. *9*, 1558.
45. Kühnemuth, B., Piseddu, I., Knott, M., Vetter, V., Layritz, P., Endres, S., and Anz, D. (2017). CCL22 impedes T cell activation capacities of dendritic cells by reducing membrane expression of MHC molecules and CD80. J. Immunol. *198*, 133.3.
46. Kim, S.-H., Park, A., Yu, J., Kim, J., Song, H.-S., Bae, J., Chae, S.Y., Kim, S., Jung, W., and Kim, Y.-M. (2021). CD82 promotes CD8+ T cell immune responses by mediating T cell polarization and immunological synapse formation. J. Immunol. *206*, 14.03.
47. Angiolilli, C., Leijten, E.F.A., Bekker, C.P.J., Eeftink, E., Giovannone, B., Nordkamp, M.O., van der Wal, M., Thijs, J.L., Vastert, S.J., van Wijk, F., et al. (2022). ZFP36 family members regulate the Proinflammatory features of psoriatic dermal fibroblasts. J. Invest. Dermatol. *142*, 402–413.
48. Korsunsky, I., Millard, N., Fan, J., Slowikowski, K., Zhang, F., Wei, K., Baglaenko, Y., Brenner, M., Loh, P.-R., and Raychaudhuri, S. (2019). Fast, sensitive and accurate integration of single-cell data with Harmony. Nat. Methods *16*, 1289–1296.
49. Aran, D., Looney, A.P., Liu, L., Wu, E., Fong, V., Hsu, A., Chak, S., Naikawadi, R.P., Wolters, P.J., Abate, A.R., et al. (2019). Reference-based analysis of lung single-cell sequencing reveals a transitional profibrotic macrophage. Nat. Immunol. *20*, 163–172.
50. Traag, V.A., Waltman, L., and van Eck, N.J. (2019). From Louvain to Leiden: guaranteeing well-connected communities. Sci. Rep. *9*, 5233.
51. Saelens, W., Cannoodt, R., Todorov, H., and Saeys, Y. (2019). A comparison of single-cell trajectory inference methods. Nat. Biotechnol. *37*, 547–554.
52. Qiu, P. (2020). Embracing the dropouts in single-cell RNA-seq analysis. Nat. Commun. *11*, 1169.
53. Cao, J., Spielmann, M., Qiu, X., Huang, X., Ibrahim, D.M., Hill, A.J., Zhang, F., Mundlos, S., Christiansen, L., Steemers, F.J., et al. (2019). The single-cell transcriptional landscape of mammalian organogenesis. Nature *566*, 496–502.
54. Trapnell, C., Cacchiarelli, D., Grimsby, J., Pokharel, P., Li, S., Morse, M., Lennon, N.J., Livak, K.J., Mikkelsen, T.S., and Rinn, J.L. (2014). The dynamics and regulators of cell fate decisions are revealed by pseudotemporal ordering of single cells. Nat. Biotechnol. *32*, 381–386.
55. Hittner, J.B., May, K., and Silver, N.C. (2003). A Monte Carlo evaluation of tests for comparing dependent correlations. J. Gen. Psychol. *130*, 149–168.
56. Bray, N.L., Pimentel, H., Melsted, P., and Pachter, L. (2016). Near-optimal probabilistic RNA-seq quantification. Nat. Biotechnol. *34*, 525–527.
57. Pimentel, H., Bray, N.L., Puente, S., Melsted, P., and Pachter, L. (2017). Differential analysis of RNA-seq incorporating quantification uncertainty. Nat. Methods *14*, 687–690.
58. Korotkevich, G., Sukhov, V., Budin, N., Shpak, B., Artyomov, M.N., and Sergushichev, A. (2021). Fast gene set enrichment analysis. Preprint at bioRxiv. https://doi.org/10.1101/060012.
59. Subramanian, A., Tamayo, P., Mootha, V.K., Mukherjee, S., Ebert, B.L., Gillette, M.A., Paulovich, A., Pomeroy, S.L., Golub, T.R., Lander, E.S., and Mesirov, J.P. (2005). Gene set enrichment analysis: a knowledge-based approach for interpreting genome-wide expression profiles. Proc. Natl. Acad. Sci. USA *102*, 15545–15550.
60. Khodadadi-Jamayran A., Pucella J., Doudican N., Carucci J., Heguy A., Reizis B., Tsirigos A. (2020). iCellR: Combined Coverage Correction and Principal Component Alignment for Batch Alignment in Single-Cell Sequencing Analysis. Preprint at bioRxiv. https://doi.org/10.1101/2020.03.31.019109.
61. Qiu, X., Mao, Q., Tang, Y., Wang, L., Chawla, R., Pliner, H.A., et al. (2017). Reversed graph embedding resolves complex single-cell trajectories. Nat. Methods *14*, 979–982.
62. Stuart, T., Butler, A., Hoffman, P., Hafemeister, C., Papalexi, E., Mauck, W.M., 3rd., et al. (2019). Comprehensive Integration of Single-Cell Data. Cell *177*, 1888–1902.e21.

STAR★METHODS

KEY RESOURCES TABLE

REAGENT or RESOURCE	SOURCE	IDENTIFIER
Antibodies		
PE anti-Human CD4	BioLegend #317410	Clone OKT4
BV711 anti-Human CD8α	BioLegend #301044	Clone RPA-T8
APC anti-Human IFNγ	BioLegend #502512	Clone 4S.B3
FITC anti-Human TNFα	BioLegend #502906	Clone MAb11
PerCP/Cy5.5 anti-Human IL-2	BioLegend #500322	Clone MQ1-17H12
TotalSeq-A anti-Human CD11c	BioLegend #371519	Clone S-HCL-3
TotalSeq-A anti-Human CD123	BioLegend #306037	Clone 6H6
TotalSeq-A anti-Human CD127	BioLegend #351352	Clone A019D5
TotalSeq-A anti-Human CD14	BioLegend #367131	Clone 63D3
TotalSeq-A anti-Human CD141	BioLegend #344121	Clone M80
TotalSeq-A anti-Human CD161	BioLegend #339945	Clone HP-3G10
TotalSeq-A anti-Human CD19	BioLegend #302259	Clone HIB19
TotalSeq-A anti-Human CD195	BioLegend #359135	Clone J418F1
TotalSeq-A anti-Human CD196 (CCR6)	BioLegend #353437	Clone G034E3
TotalSeq-A anti-Human CD197 (CCR7)	BioLegend #353247	Clone G043H7
TotalSeq-A anti-Human CD1c	BioLegend #331539	Clone L161
TotalSeq-A anti-Human CD21	BioLegend #354915	Clone Bu32
TotalSeq-A anti-Human CD207 (Langerin)	BioLegend #352207	Clone 10E2
TotalSeq-A anti-Human CD25	BioLegend #302643	Clone BC96
TotalSeq-A anti-Human CD294	BikoLegend #350127	Clone BM16
TotalSeq-A anti-Human CD3	BioLegend #300475	Clone UCHT1
TotalSeq-A anti-Human CD4	BioLegend #344649	Clone SK3
TotalSeq-A anti-Human CD45RA	BioLegend #304157	Clone HI100
TotalSeq-A anti-Human CD45RO	BioLegend #304255	Clone UCHL1
TotalSeq-A anti-Human CD56 (NCAM)	BioLegend #362557	Clone 5.1H11
TotalSeq-A anti-Human CD69	BioLegend #310947	Clone FN50
TotalSeq-A anti-Human CD8	BioLegend #344751	Clone SK1
TotalSeq-A anti-Human CD80	BioLegend #305239	Clone 2D10
TotalSeq-A anti-Human HLA-DR	BioLegend #307659	Clone L234
Biological samples		
Human Peripheral Blood Leukopak, Fresh		Cat# 200-0092
Chemicals, peptides, and recombinant proteins		
Recombinant Human IL-2	PeproTech	Cat# 200-02
X-VIVO 15 Serum-free Hematopoietic Cell Medium	Lonza	Cat# BE02-060Q
FBS	UCSF Cell Culture Facility	Cat# CCFAQ008
DAPI	Life Technologies	Cat# A20502
Recombinant Cas9-NLS	UC Berkeley QB3 MacroLab	No Cat#
Dynabeads Human T-Activator CD3/CD28	ThermoFisher Scientific	Cat# 11132D
PBS	UCSF Cell Culture Facility	Cat# CCFAL003
β-Mercaptoethanol	ThermoFisher Scientific	Cat# 21985023
RPMI-1640	UCSF Cell Culture Facility	Cat# CCFAE001
DNAse I	Sigma	Cat# 10104159001
Collagenase IV	Worthington Biochemical Corp.	Cat# LS004188

(*Continued on next page*)

Continued

REAGENT or RESOURCE	SOURCE	IDENTIFIER
Penicillin/Streptomycin	ThermoFisher Scientific	Cat# 15140122
HEPES	UCSF Cell Culture Facility	Cat# CCFGL001
Human TruStain FcX	BioLegend	Cat# 422301
Cell Stain Buffer	BioLegend	Cat# 420201
SPRI beads	Beckman-Coulter	Cat# B23317
PMA/Ionomycin	BioLegend	Cat# 423301
Brefeldin A	BioLegend	Cat# 420601
Monensin	BioLegend	Cat# 420701
Critical commercial assays		
Kapa library quantitation kit	Kapa Biosystems	Cat# KK2601
Chromium Single cell 3′ Solution V3 kit	10x Genomics	Cat# 2000059
Phusion High-Fidelity DNA Polymerase	ThermoFisher Scientific	Cat# F-530XL
P3 Primary Cell 4D-Nucleofector X Kit S	Lonza	Cat# V4XP-3032
Chromium Single cell 3′ Solution V2 kit	10x Genomics	Cat# 220104
Deposited data		
scRNA-seq BAM files	This study	European Genome-Phenome Archive (EGA) accession number EGA: S00001005271
RNA-seq FASTQ files	This study	European Genome-Phenome Archive (EGA) accession number EGA: S00001005271
Oligonucleotides		
PCR Primers and gRNA sequences	Integrated DNA Technologies	EGA: S00001005271. See Table S6
Software and algorithms		
R version 4.0.5	R Foundation	https://www.r-project.org
Cell Ranger 3.0.2	10X Genomics	https://support.10xgenomics.com/single-cell-gene-exp
Seurat 4.0.2	Stuart et al., 2019[62]	https://github.com/satijalab/seurat
monocle3 1.2.7	Qiu et al., 2017[61]	https://cole-trapnell-lab.github.io/monocle3/docs/introduction/
Harmony	Korsunsky et al., 2019[48]	https://github.com/immunogenomics/harmony
ggplot2 3.3.3	Hadley Wickham	https://github.com/tidyverse/ggplot2
KallistoK 0.46.1	Bray et al., 2016[56]	https://pachterlab.github.io/kallisto/manual
sleuth 0.30.0	Pimentel et al., 2017[57]	https://pachterlab.github.io/sleuth/
fgsea 1.12.0	Korotkevich et al., 2021[58]	https://bioconductor.org/packages/release/bioc/html/fgsea.html
MSigDB 7.4	Subramanian et al., 2005[59]	http://www.gsea-msigdb.org/gsea/msigdb/index.jsp
singleR 3.13	Aran et al., 2019[49]	https://github.com/dviraran/SingleR
muscat 1.10.1	Crowell et al., 2020[12]	https://github.com/HelenaLC/muscat
iCellR 1.6.4	Khodadadi-Jamayran et al., 2020[60]	https://github.com/rezakj/iCellR

RESOURCE AVAILABILITY

Lead contact

Correspondence and request for materials should be addressed to the lead contact for resources, Dr. Jeffrey B. Cheng (Jeffrey.Cheng@ucsf.edu).

Materials availability

This study did not generate new unique reagents.

Data and code availability

- Sequence data is submitted at the European Genome-Phenome Archive (EGA), which is hosted by the EBI and the CRG, under accession number EGA: S00001005271. Flow cytometry data reported in this paper will be shared by the lead contact upon request.
- All analysis scripts are available at the online repository https://github.com/cpcook1/TTP. All statistical analysis and plotting of scRNA-seq and cell surface protein data were performed using Rstudio software (v1.2.5033).
- Any additional information required to reanalyze the data reported in this paper is available from the lead contact upon request.

EXPERIMENTAL MODEL AND SUBJECT DETAILS

Patient characteristics

Normal, healthy control skin obtained from patients undergoing mastectomies or reduction abdominoplasties and psoriasis lesional skin were obtained using protocols approved by the UCSF Human Research Protection Program Institutional Review Board. Informed consent was obtained from all patients donating samples based on these active protocols. Adult patients donating psoriasis samples from the trunk or proximal extremity were assessed at a Psoriasis Area Severity Index (PASI score) of 8 or more (moderate to severe disease). Histopathology was verified by a board-certified dermatopathologist. Patients undergoing IL23 blockade (tildrakizumab) treatment were enrolled under an IRB protocol as part of a study funded by Sun Pharma, had a pre-treatment PASI score of 7 or greater, and received at least two 100 mg treatments before mid-treatment biopsy of clinically resolved lesions at > 8 weeks. Comprehensive biographical metrics of all subjects from this study are available in Table S1.

METHOD DETAILS

Skin sample collection, single cell RNA-seq, and CITE-seq processing

Skin samples were obtained by 6 mm punch biopsy and minced into fine pieces using surgical scissors and transferred into 3 ml of RPMI-1640 medium supplemented with 10% fetal bovine serum, 100 IU/mL Penicillin+100 μg/mL Streptomycin, 10mM HEPES, collagenase type IV (200U/ml; Worthington) and 20 μg/ml DNAse I (MilliporeSigma), then incubated for 16-18h at 37°C with 5% CO_2. After incubation, the suspension was filtered through a 100 μm cell strainer, pelleted by centrifugation at 1500 rpm for 5 min, and resuspended in 100 μL of FACS buffer (PBS supplemented with 2% FBS) containing APC conjugated human CD45 Antibody at a 1:33 dilution. After staining at 4°C for 30 min, cells were washed twice in FACS buffer, passed through a 45 μm filter and sorted for live $CD45^+$ cells following the addition of DAPI at 1 μg/ml. For samples where CITE-seq was performed (Table S2), murine splenocytes were added to comprise 5% of total cells (to allow for computational adjustment for non-specific antibody binding). Blocking was performed with the addition of 5% Human TruStain FcX (Biolegend) in a 100 μL cell suspension volume for 10 minutes at 4°C, followed by CITE-seq antibody addition for 30 min at 4°C (TotalSeq-A antibodies, 0.5 ug for each antibody per 1 million cells; Biolegend: Table S2). Cells were washed three times in Cell Stain Buffer (BioLegend) and concentrated by pelleting and resuspended in a smaller volume prior to Chromium chip loading. Single-cell RNA-sequencing libraries were then prepared from isolated cells using the Chromium Single cell 3' Solution V2 or V3 kit (10x Genomics) following the manufacturer's protocol by the Genomics Core Facility UCSF Institute for Human Genetics (Table S2). For CITE-seq samples, 0.2 pmol of ADT (antibody derived tag) additive primer was added at the RNA library cDNA amplification step. CITE-seq libraries were prepared according to the manufacturer's TotalSeq-A antibody protocol (BioLegend). In brief, 70 μL of ADT-containing cDNA amplification supernatant was purified with two rounds of 2X SPRI beads (Beckman-Coulter) and then amplified for 14-20 cycles using HiFi HotStart ReadyMix (KAPA, Roche Sequencing & Life Science) and 0.25 μM of oligos corresponding to SI PCR primer and Truseq Small RNA RPI1-6 primers. The resulting amplification products were purified by 1.2X SPRI bead cleanup, then quantified with Qubit dsDNA HS Assay Kit. Quality for scRNA and ADT libraries was assessed by a TapeStation D1000 ScreenTape (Agilent Technologies Inc.) and quantitated by the Kapa library quantitation kit prior to sequencing. mRNA and ADT libraries were sequenced on a HiSeq 4000 (Illumina) with a read length configuration of 150 PE.

Single cell RNA sequencing data processing

FASTQ files derived from sequencing were aligned and quantified using Cell Ranger Software (v3.0.2, 10x Genomics) against the human GRCh38 reference transcriptome. A filtered data matrix was used which excludes empty droplets and doublets. Quality of cells were then assessed based on the total number of detected genes per cell and the percentage of mitochondrial gene counts. Cells were filtered if there were unique feature counts greater than 5000 or mitochondrial gene counts higher than 20%. Normalized counts were then computed based on feature counts for each cell divided by the total counts for that cell and multiplied by the scale factor (1e4) with natural-log transformation. Finally, about 500 to 6,000 cells per sample were retained for downstream analysis (Table S2). Based on these criteria, high-quality transcriptomic data was obtained from 68,835 single cells after removal of doublets and cells with high mitochondrial content or low number of genes. The median gene count per cell was 1351 post-filtering and the average total number of detected genes per donor was 18,048 (detailed sequencing metrics for each sample are in Table S2).

Dimensional reduction and unsupervised clustering

The harmony algorithm[48] was used for batch effect correction and integration of cells into a coordinated space for unsupervised clustering. To detect high variable features (HVFs) used for harmony, the variance stabilizing transformation parameter was used as a function input. After scaling the data, a PCA matrix was calculated using detected HVFs with 20 components based on the ElbowPlot function in Seurat v4.0.2. We then provided this PCA matrix to the RunHarmony() function in Seurat using samples as technical covariates for correction. The batch-corrected coordinated space then was used to compute the nearest neighbor graph by the FindNeighbors() function.

Indeed, across cell types prior to batch correction, samples clearly fell out as coherent clusters in the UMAP (Figure S1D), indicating that a large proportion of transcriptome-wide variation correlated to sample. However, upon batch correction, sample-specific cells no longer cohered but disease-level clusters continued to be clear (Figure S1D). We therefore concluded that sample-level batch correction successfully eliminated potential sample-level confounding while preserving target disease-level variation.

This nearest neighbor graph was used by FindClusters() using the Louvain algorithm for clustering of cell populations and dimensional reduction such as UMAP and tSNE. Based on clustree optimization, we utilized resolution = 0.4 to obtain 14 initial immune clusters (6 *CD3*$^+$ or *KLRB1*$^+$ lymphocyte and 8 *HLA-DRA*$^+$ antigen presenting cell clusters). We grouped the lymphocyte cell clusters independently and further subclustered them into 21 *CD3*$^+$ or *KLRB1*$^+$ clusters to achieve finer resolution. To filter out cells containing markers previously identified as associated with collagenase digestion, and thus representing potential artifacts[41], we removed clusters 15 and 21 (as numbered in the Seurat object), which were highly enriched for *JUN*, *FOS*, *HSPA1B*, and *HSP1B*. We retained for analysis the six major T cell populations that each represented more than 5% of all T cells from our sample dataset. Cluster-specific differentially expressed genes were detected using the FindMarkers() function in the Seurat package using the MAST method and manually annotated based on the expression of known marker genes and then were checked using the singleR package v3.13.[49] The Human Primary Cell Atlas was used as a reference dataset for annotation.

To identify genes differentially expressed between psoriasis and matched normal samples, we developed pseudo-bulk counts for each cluster in each patient using the Bioconductor package *muscat*[12], which then applies *edgeR* to detect statistically significant differences between the patient groups.[13]

To deconvolute CD4$^+$ and CD8$^+$ T cells in the Trm1 population, we took a conservative transcriptional gating approach to ensure that we called high-confidence helper and cytotoxic T-cells. We used empiric unimputed data, filtering out non-expressing or double positive cells (double positive were defined as cells expressing *CD4*/*CD8A* and/or *CD4*/*CD8B*). We then defined *CD4*$^+$ cells as those expressing detectable *CD4* transcripts and *CD8*$^+$ cells as those expressing either *CD8A* or *CD8B* transcripts. For analysis in Figure 6, all cells containing at least one *IL17A*$^+$ or *IL17F*$^+$ read were counted and then corrected for sampling of the overall. The correction factor was (total CD45$^+$ cells isolated in the biopsy/total CD45$^+$ cells profiled by scRNA-seq).

scRNA-seq correlation analysis

To identify genes associated with *ZFP36L2* expression, we used Spearman rank correlations to search through cluster-specific sets of high variability genes. First, we identified the top 5000 variable genes across cells within sample- and cluster-specific subpopulations using the Seurat function FindVariableFeatures. Within these cell subpopulations, we then tested for the association between *ZFP36L2* and each of these highly variable genes with Spearman correlations implemented in the cor.test function from the Stats package v3.6.2. We accounted for multiple testing by adjusting p-values with the Benjamini-Hochberg procedure.

For Figures 2A, 2B, and 4A, the standard imputed expression value shown on the *x*-axis represents count data that has been normalized and log-transformed by R/Seurat's default standardization and normalization algorithms, and imputed by R/iCellR/ run.-impute(my.obj, dims = 1:10, nn = 10, data.type = pca).

Trm1 manifold and developmental trajectory inference

We analyzed Trm1 subpopulation cells with the goal of understanding how unbiased developmental trajectories could be inferred from them, using these spatially separate subjects as a space-for-time substitutions (*i.e.* how cells could develop from uninflamed to inflamed states using samples separated in space among different subject to infer temporal transitions in pseudotime). Since human subjects differ in a number of potentially confounding ways, we controlled for sample-level variation by implementing a mutual nearest neighbor batch correction routine to 'regress out' the effect of sample-specific variation, presuming that the residual variation would be produced by disease-level rather than sample-level variation.[59] Indeed, this proved to be the case since disease correlated strongly to axes 1 and 2 of the resulting uMAP, indicating that the plurality of variation in the data after accounting for sample-specific variation correlated to disease state. We then undertook a manifold optimization routine to balance local and global structure in the data based on: 1) global connectedness to support the inference of a global trajectory and 2) local clustering to isolate specific disease and cell-state subpopulations. First, we re-composed Trm1 cells in a uMAP, exploring 3,000 combinations in Nn (number of nearest neighbors [5,50] and Md (minimum nearest neighbor distance [0.1,0.9], finding 10 and 0.8 respectively to strike the best balance between global and local information on manual inspection. We then used Leiden-based clustering at default resolution (k=10, partition q-value=0.05) to call unsupervised clusters for downstream lineage inference.[50]

Since the true branching structure and hierarchy in Trm1 cells are unknown, we used Monocle3 to infer a rooted trajectory since it has been benchmarked in controlled trials as the most informative and robust method to interrogate complex developmental topologies in single cells.[51] We then used the Monocle3/learn_graph function without partition-consideration to infer a global trajectory.[52,53] We then

overlaid clinical information on the uninflamed or inflamed provenance of each cell onto the manifold, knowing that the true trajectory begins with uninflamed cells. Node 1 occurred in a highly dense cluster of uninflamed cells oriented along axes 1 and 2 away from the majority of inflamed cells, and thus, we rooted our global trajectory in this node and calculated pseudotemporal ordering from it using the order_cells function in Monocle3.[54] We then observed that pseudotemporal order and *ZPF36L2* expression were broadly correlated to Axis 1 and testing for the Spearman rank correlation between them revealed a significant anticorrelation. Since this result concords with inflammation-associated downregulation in *ZPF36L2* observed in clinical specimens, we considered this topology to reflect true *ZFP36L2*-associated dynamics in these cells. We then repeated these associations by integrating over specific sets of genes using the aggregate_gene function in Monocle3 without maximum or minimum cutoffs. These scaled, integrated gene expression values enabled the direct comparison of the total expression of sets of genes among cells with a single value, which were overlaid on the manifold and correlated with pseudotime as in Figure 5. The association between the integrated expression of these programs and pseudotime were not linear and thus we fit LOESS curves to visualize overall trends.

Combinatorial single-cell inflammatory mediator prediction analysis

Since the aggregation of gene sets smoothed over gene-level variation within these sets, it was not clear which constituent genes within a set may be driving correlational patterns observed in Figure 6. In order to test this directly, we implemented a combinatoric approach to test the correlation of all possible, non-repeating combinations of genes of all set sizes between predictive sets and single response genes. The predictive set consisted of *ZFP36L2*, *ZFP36*, *CCL22*, *SOCS1*, *SOCS3*, *BTG1*, *TXNIP*, *CD69*; and the response genes were *IL17A*, *IL17F*, *IL26*, *IFNG*, *CXCL13*, *CD2*, *CD3E*, *CD3G*, *CD82*, *TFRC*, *LAYN*, *GNLY*, *ENTPD1*, *CPM*, *NPDC1*, *ZEB2*. Gene values were batch-corrected at the sample level using the CPCA method in the R package iCellR; missing gene values were independently imputed within inflamed and unflamed states of sample-aligned matrices using the PCA method in iCellR/run.impute. Resulting matrices were then passed to Monocle3, and all possible combinations of predictor genes were then summed using the aggregate_gene function, and their correlation with response genes calculated with Spearman rank correlations using the cor.test function.

To develop pairwise statistical tests of the predictive power of these *ZIST* gene combinations, we developed a stepwise model comparison algorithm using R/cocor. We used this package to implement a two-sided test between the correlations of independent variables (in this case the gene sets). This method relies on Hittner, May, and Silver's modification of Dunn and Clark's Z statistic[55], which in turn uses a backtransformed Fisher's Z procedure. To control type 1 error by shrinking the test space, we ordered pairwise steps according to correlation coefficients of gene sets and tested for correlation differences only between two adjacent gene sets.

Preparation of CRISPR/CAS9 Ribonucleoprotein particles

Five gRNAs, two each for *ZFP36L2* and *ZFP36* and one targeting the *AAVS1* locus with the following sequences were utilized:

L2g1: 5'- CGCCGTTCTCGCTAAACGAG -3'
L2g2: 5'- CTGCCACTTCATCCACAACG – 3'
36g1: 5' - GCTACAAGACTGAGCTATGT -3'
36g2: 5' - CAACCCTAGCGAAGACCTGG – 3'
AAVS1: 5' – GGGACCACCTTATATTCCCA-3'

gRNAs and trans-activating crRNA (tracrRNA,IDT) were resuspended to 160 μM in Duplex Buffer (IDT technologies), mixed in equimolar quantities, heated to 95°C for 5 min and cooled slowly to room temperature to facilitate annealing of the oligomers. Polyglutamic acid (15-50 kDa) was added to a final concentration of 2 mg/mL followed by addition of Cas9-NLS (QB3 MacroLab, University of California, Berkeley) to a final concentration of 10 μM. The ribonucleoprotein particles (RNPs) were incubated for 15 min at 37 °C then stored at 4°C prior to nucleofection the same day.

Donor T cell nucleofection

T cells were isolated from donor leukopaks (STEMCELL Technologies) by negative selection with CD3 magnetic beads (EasySep Human T Cell Isolation Kit; STEMCELL Technologies) according to the manufacturer's protocol and frozen in LN_2 at $20x10^6$ cells/mL for storage. Donor T cells were then thawed, washed twice in X-VIVO 15 media (Lonza) then resuspended at $1x10^6$ cells/mL in T cell medium (X-VIVO 15 supplemented with 5% FBS, 55 μM 2-mercaptoethanol and 200 IU/mL recombinant human IL2) prior to the addition of CD3/CD28 conjugated magnetic activation beads (Dynabeads; Invitrogen) at a 1:1 bead-to-cell ratio. Forty-eight hours later the cells were washed 1x in PBS, resuspended in 20 μL of supplemented P3 nucleofection solution (Lonza) at $50x10^6$ cells/mL, and immediately added to the RNPs and mixed by gentle pipetting. The cell/RNP mixture was placed in one well of a 16-well cuvette strip (P3 Primary Cell 4D-Nucleofector X Kit S; Lonza) and electroporated using program EH115 on a Lonza X-Unit nucleofector. Immediately after, 100 μL of warm X-VIVO+5% FBS was added to the well, and the cells were placed in a 5% CO_2 incubator at 37°C for 15 min, then plated in 4 mL of T cell medium. Cells were monitored daily and split 1:1 when the density reached $2x10^6$ cells/mL or media showed signs of acidification.

PCR-based validation of CRISPR-based T cell gene knockout

Genomic DNA was extracted from edited primary T cells seven days after nucleofection using a Wizard Genomic DNA Purification Kit (Promega). A ~500 bp fragment flanking the CRISPR/Cas9 cut site for each knockout and gene was then amplified by PCR, utilizing the following primer pairs:

L2g1-F – 5’ CTCAACCTGAACAACATGC 3’
L2g1-R – 5’ TGTACTTCGGATGGCGA 3’
L2g2-F 5’ AGCGAGAACGGCGATC 3’
L2g2-R 5’ GGCTGTCGAGCAGCA 5’
36g1+36g2-F 5’ GCTCCACCAGCCTAGTGG 3’
36g1+36g2-R 5’GGGTCTCTTCGAGCCAGG 5’
AAVS1-F 5’ TCCTGTGGATTCGGGTCA 3’
AAVS1 -R 5’ GCTCCATCGTAAGCAAACCT 3’

PCR amplifications were carried out using Phusion High-Fidelity DNA Polymerase (ThermoFisher) with reagent concentrations specified in the product catalog. Thirty-five cycles were run with a denaturation time of 30 s at 98°C, followed by an annealing step for 30 s at varying temperatures to match specific primer Tms, followed by an elongation step at 72°C for 30 s. Single band PCR products were purified using a PCR Purification Kit (Qiagen) while PCR products with more than one band were gel extracted (Qiagen Gel Purification Kit). Edited samples and non-targeting controls underwent Sanger sequencing (Quintara Biosciences) utilizing a primer specific for the target locus. Resulting AB1 files were imported into the online ICE algorithm portal (Synthego) and assessed for knockout efficiency.

Intracellular cytokine assays of CRISPR-modified T cells

T cells were seeded in 200 μL of X-VIVO supplemented with 5% FBS in a 96-well U-Bottom plate at 2.5×10^6 cells/mL. PMA + ionomycin resuspended in DMSO was added to give final concentrations ranging from 50 pg/mL to 50 ng/mL PMA and 0.1 ng/mL to 10 ng/mL ionomycin and the cells were incubated for 5 h at 37°C. After 1 h, 1 μL each of a 1:5 dilution of Brefeldin A (BioLegend) and Monensin (BioLegend) in X-VIVO+5%FBS was added. The cells were then washed 1X in PBS and resuspended in Zombie Aqua Live/Dead (Biolegend) diluted 1:100 in PBS and incubated at room temperature for 15 min in the dark. Cells were then washed 2X in FACS buffer and resuspended in 100 μL of FACS buffer containing 1:100 dilutions of both PE αHuman CD4 Clone OKT4 (Biolegend #317410) and BV711 αHuman CD8 Clone RPA-T8 (Biolegend #301044), then incubated for 20 min at 4°C. Cells were then fixed and permeabilized (CytoFix/CytoPerm; BD) according to manufacturer’s instructions and resuspended in 100 μL Perm/Wash buffer containing 1:40 dilutions Abs to the following intracellular cytokines: APC αHuman IFNγ clone 4S.B3 (Biolegend #502512), FITC αHuman TNFα clone MAb11(Biolegend #502906) and PerCP/Cyanine5.5 αHuman IL-2 clone MQ1-17H12 (Biolegend #500322). Samples were then washed and data was collected on an Attune Nxt Cytometer (UCSF LCA Core) and analyzed with FlowJo (BD).

mRNA isolation and bulk RNA-seq of CRISPR-modified CD4$^+$ T cells

Nucleofected T cells as described above were washed 1x in FACS buffer then stained with cell surface antibodies to CD4 (PE Human CD4 Clone OKT4; Biolegend #317410) for 30 min at 4°C. After washing unbound antibody, DAPI was added at 10 μg/mL and CD4$^+$ cells were sorted into X-VIVO+20% FBS after gating for live singlets. The cells were then washed in X-VIVO supplemented with 5% FBS and 55 μM 2-mercaptoethanol and were incubated in a 5% CO_2 incubator at 37°C. On the day of the experiment, 1×10^6 cells were plated in 1mL of X-VIVO media supplemented with 5% FBS and 0.5 ng/mL PMA+10 ng/mL ionomycin for 4 h in a CO_2 incubator at 37°C. Following incubation, the cells were pelleted and resuspended in 750 μL TRIzol Reagent (Invitrogen). RNA extraction and DNAse treatment was carried out using a Direct-zol RNA Miniprep kit (Zymo Research) according to manufacturer’s instructions. RNA sample quality was assessed by the Bioanalyzer Pico kit (Agilent Technologies Inc.) and quantified by Qubit 2.0 RNA HS assay (ThermoFisher). Total RNA was combined with paramagnetic beads coupled with oligo d(T)25 to isolate poly(A)+ transcripts using the NEBNext® Poly(A) mRNA Magnetic Isolation Module (New England BioLabs Inc.). Prior to first strand synthesis, samples were randomly primed and fragmented per manufacturer’s recommendations. The first strand was synthesized with the Protoscript II Reverse Transcriptase for 30 min at 42°C. All remaining steps for library construction were performed per the manufacturer’s protocol for the NEBNext UltraTM II Non - Directional RNA Library Prep Kit for Illumina (New England BioLabs Inc.). Libraries were quantified by Qubit 2.0 (ThermoFisher) and quality assessed by TapeStation HSD1000 ScreenTape (Agilent Technologies Inc). Average final library size was about 400 bp with an insert size of about 280bp. Illumina 8-nt dual-indices were used. Equimolar pooling of libraries was performed based on QC values and sequenced on an Illumina NovaSeq S4 (Illumina) with a read length configuration of 150 PE for 40 M PE reads per sample (20M in each direction).

Bulk RNA-seq analysis

RNA transcript abundance was quantified with KallistoK version 0.46.1[56] using 100 bootstraps and a prebuilt index based on Ensemble v96 from https://github.com/pachterlab/kallisto-transcriptome-indices. Differential expression (DE) analysis was performed with sleuth version 0.30.0[57] in gene mode. Likelihood-ratio tests were used to compare sleuth models fit with both treatment and donor as covariates against models that only used the donor covariate. Genes were selected for DE testing based on a threshold of log2(normalized TPM +1) > 1 in at least half of the samples. Of the genes passing the expression filter, the 2000 genes with highest log-TPM variance were used. We performed gene set enrichment analysis using fgsea v1.12.0[58] with a max gene set size of 500 and 100,000 permutations. 2922 gene sets from canonical pathways in the curated gene sets collection from MSigDB v7.4[59] were used in the enrichment analysis.

QUANTIFICATION AND STATISTICAL ANALYSIS

Statistical analysis for flow cytometry data

Data is presented as mean ± SEM Statistical differences between groups were calculated with Student's two-sample, two-tailed, homoscedastic *t*-test as outlined in the Figure Legends. Significance is denoted by *$p < 0.05$, **$p < 0.01$, ***$p < 0.001$, ****$p < 0.0001$.

Statistical analysis for scRNA-seq and bulk RNA-seq data

Detailed and comprehensive statistical parameters underlying the analysis of scRNA-seq and bulk RNA-seq data can be found in Method details and corresponding figure legends.

Cell Reports Medicine, Volume *3*

Supplemental information

A single-cell transcriptional gradient in human cutaneous memory T cells restricts Th17/Tc17 identity

Christopher P. Cook, Mark Taylor, Yale Liu, Ralf Schmidt, Andrew Sedgewick, Esther Kim, Ashley Hailer, Jeffrey P. North, Paymann Harirchian, Hao Wang, Sakeen W. Kashem, Yanhong Shou, Timothy C. McCalmont, Stephen C. Benz, Jaehyuk Choi, Elizabeth Purdom, Alexander Marson, Silvia B.V. Ramos, Jeffrey B. Cheng, and Raymond J. Cho

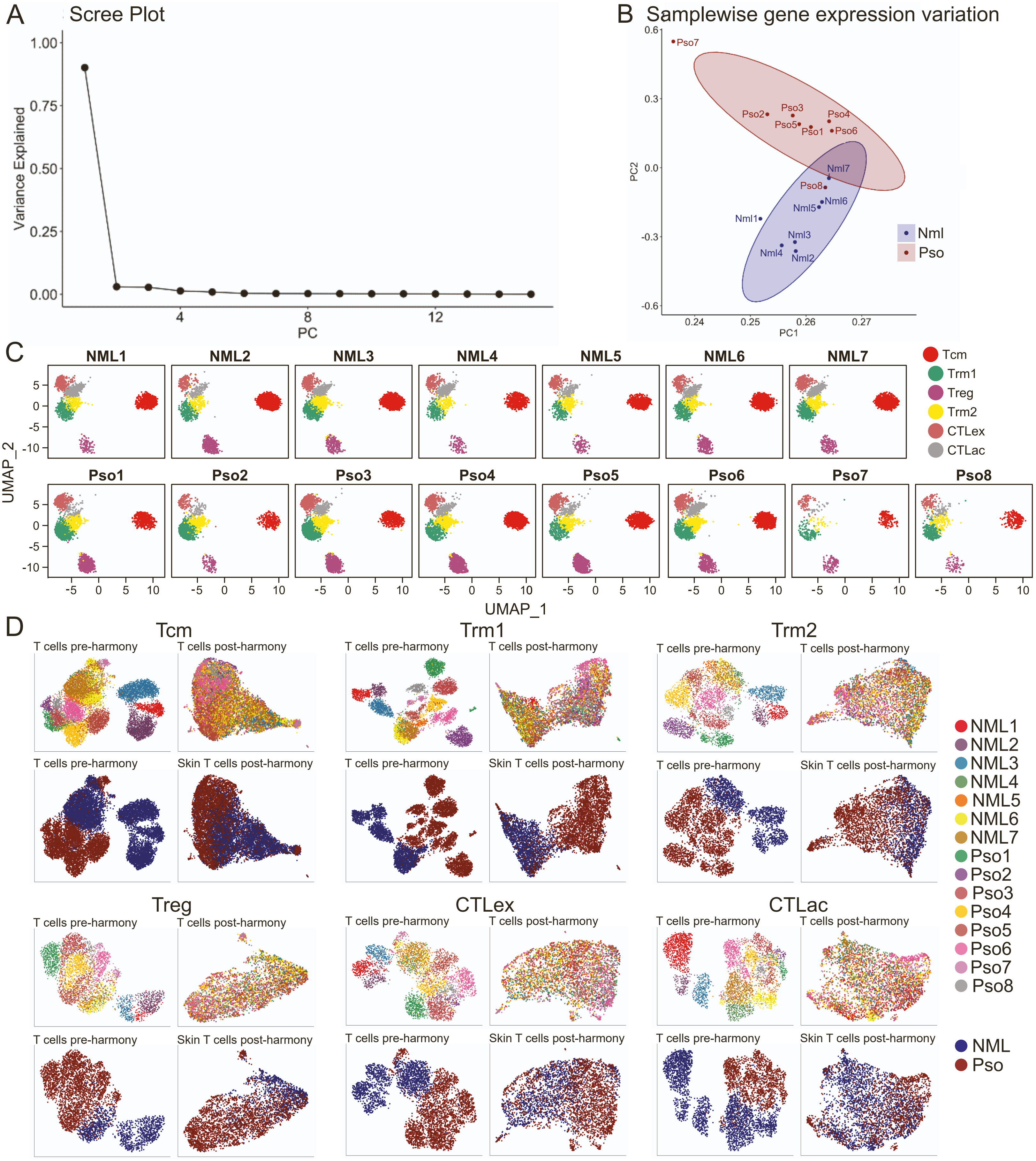

Figure S1 - Illustration of biological data consistency across patient samples (Related to Figure 1). A) Scree plot of eigendecomposition showing that PC1 and PC2 explain 93.4% of variation in gene expression (all 15 samples). B) Plot of all samples vs. PCs 1 and 2 illustrating that normal (Nml) and psoriasis (Pso) samples cluster coherently in PC space, with sparse overlap in their respective 95%-confidence bivariate ellipses. C) uMAP graphs showing T cell populations from each Nml and Pso sample. While Main Figure 1B shows each cluster derives substantial representation from each healthy and normal sample, Figure S1C illustrates how the distinct identity of each cluster is broadly replicated in each sample. D) uMAP representations of each major T cell cluster before and after batch correction (all 15 samples aggregated), illustrating necessity for correction.

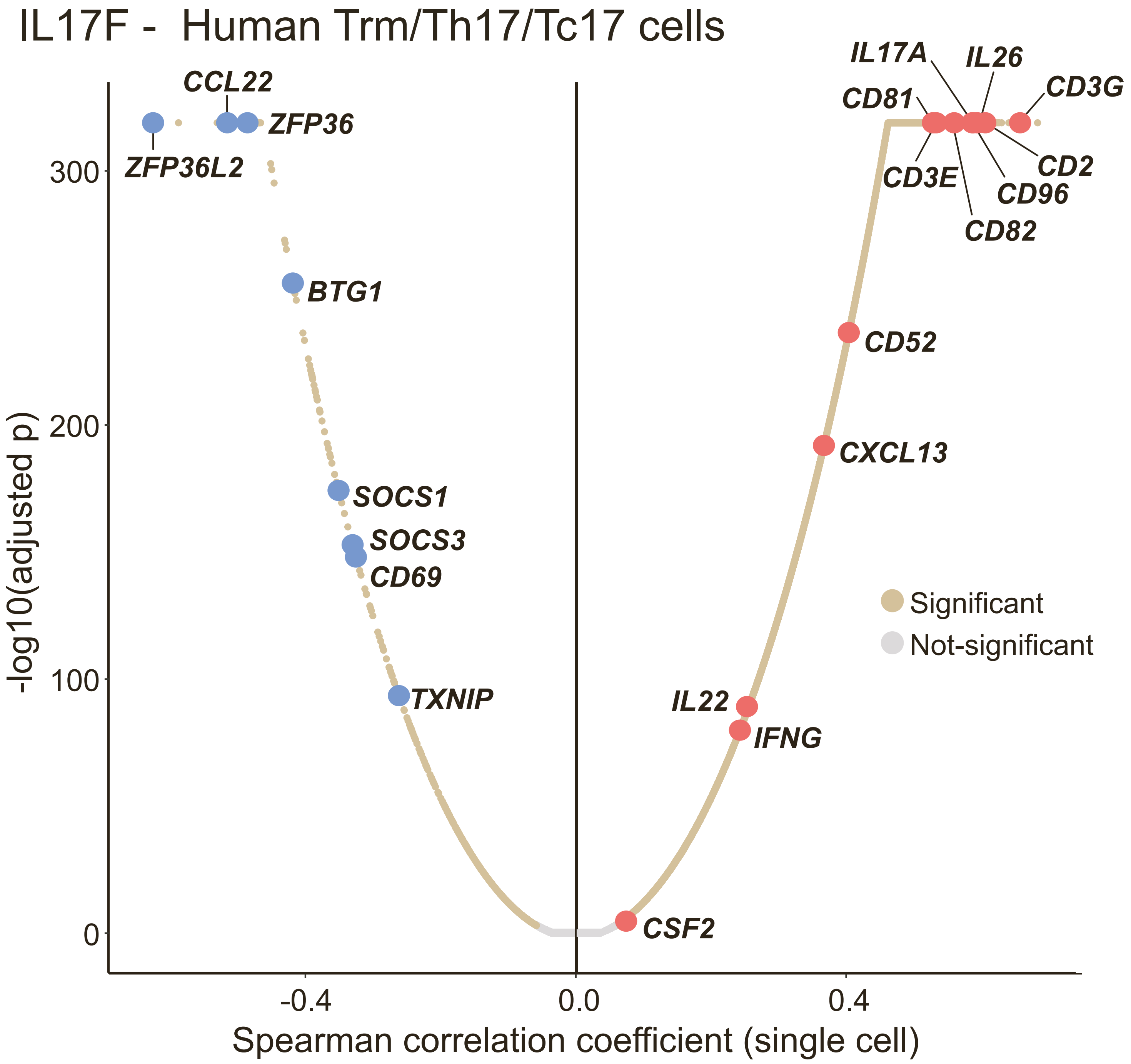

Figure S2 - Enrichment in *IL17F*-expressing single skin Trm1 cells for expression of pro-inflammatory transcripts and cytokines (Related to Figure 2). Positive correlation of *IL17F* expression in single Trm1 cells with expression of pro-inflammatory transcripts and cytokines (right half of graph, labelled in red). Deficiency in *IL17F*-expressing single Trm1 cells of inflammation-suppressive genes such as *ZFP36L2*, *ZFP36*, and *BTG1* is shown as negative Spearman correlations (left half of graph, labelled in blue). Data is shown for all 8 psoriasis and 7 normal samples.

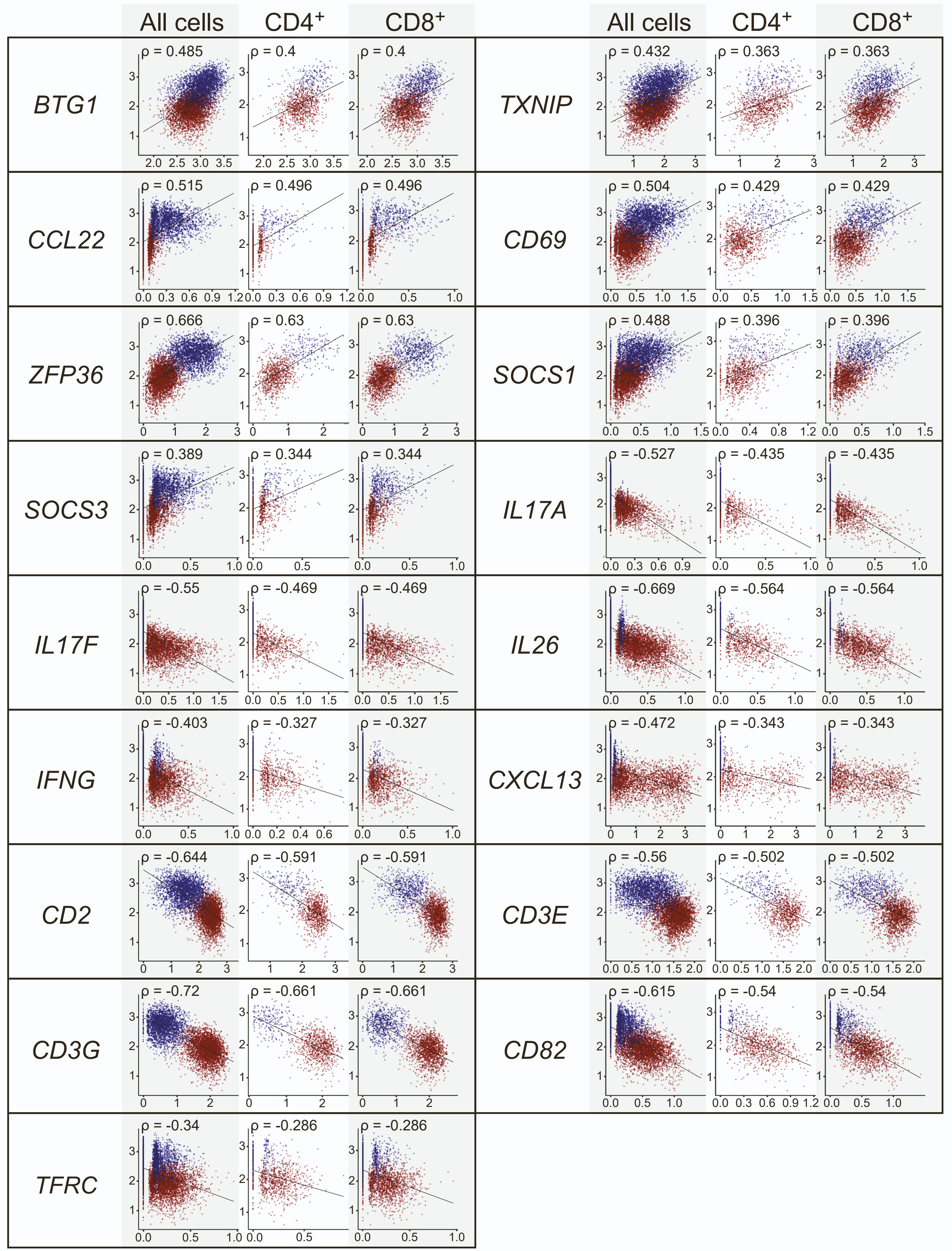

Figure S3 – *ZIST* gradient is detected in both Th17 and Tc17 subpopulations (Related to Figure 2). *ZFP36L2* positive and negative correlations with *ZIST* genes and Th17/Tc17 cytokines are shown to persist in both CD4+ (i.e. Th17) and CD8+ (i.e. Tc17) single cell subpopulations when separated based on expression of one or the other transcript (double negative and double positive cells were excluded, red shows all 8 psoriasis samples pooled and blue shows all 7 healthy controls pooled). y-axis shows *ZFP36L2* expression, x-axis shows imputed cytokine transcript levels, each point represents a single skin-resident T cell.

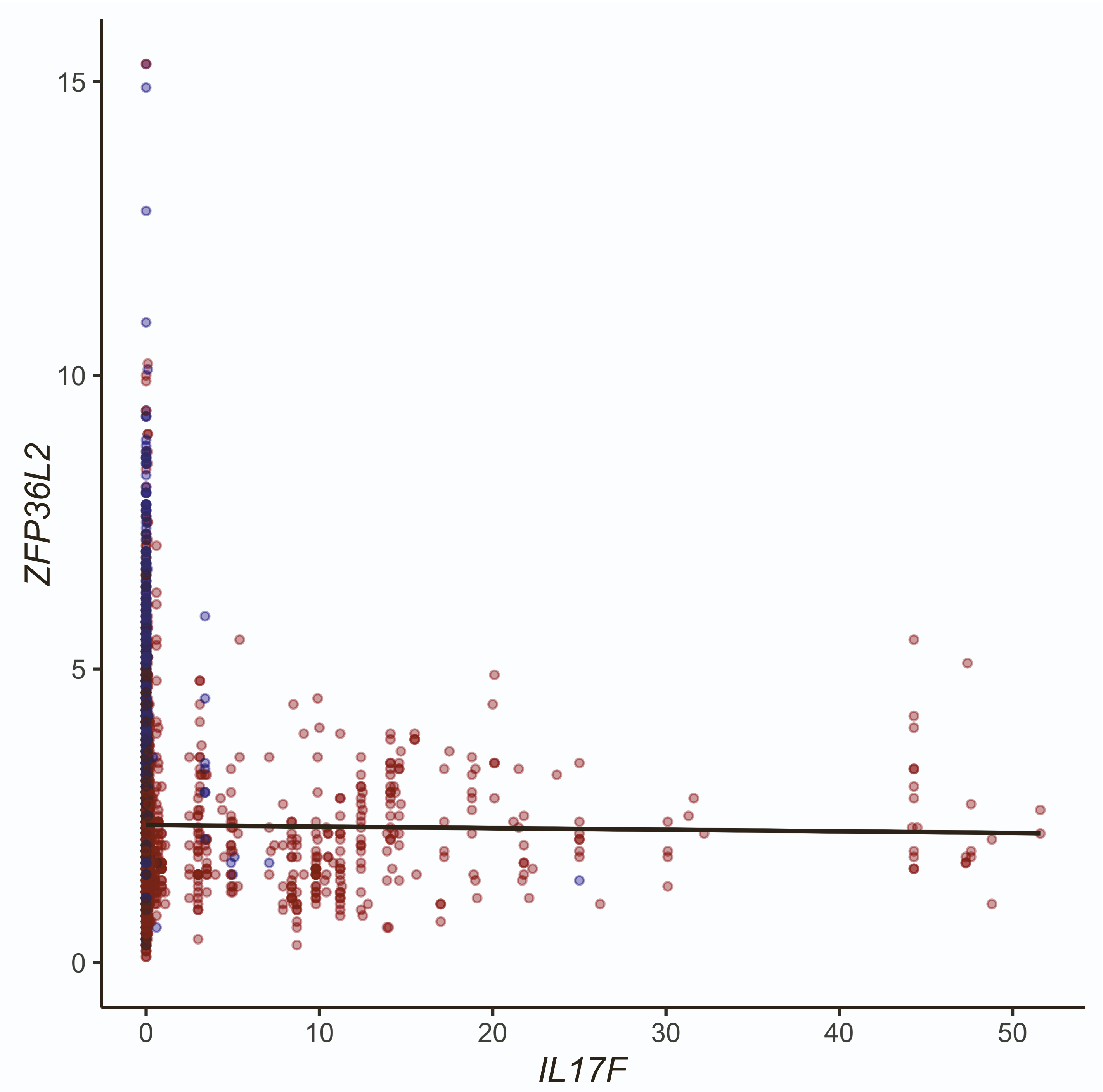

Figure S4 - Suppression of *IL17F* in *ZFP36L2*-expressing human CD4+ PBMCs (Related to Figures 2, 4). Relationship between *IL17F* (x-axis) and *ZFP36L2* (y-axis) expression in CD3/CD28 bead-activated (red) and naïve (blue) T cells from Cano-Gamez *et al*, imputed transcript levels shown, each point represents a single T cell.

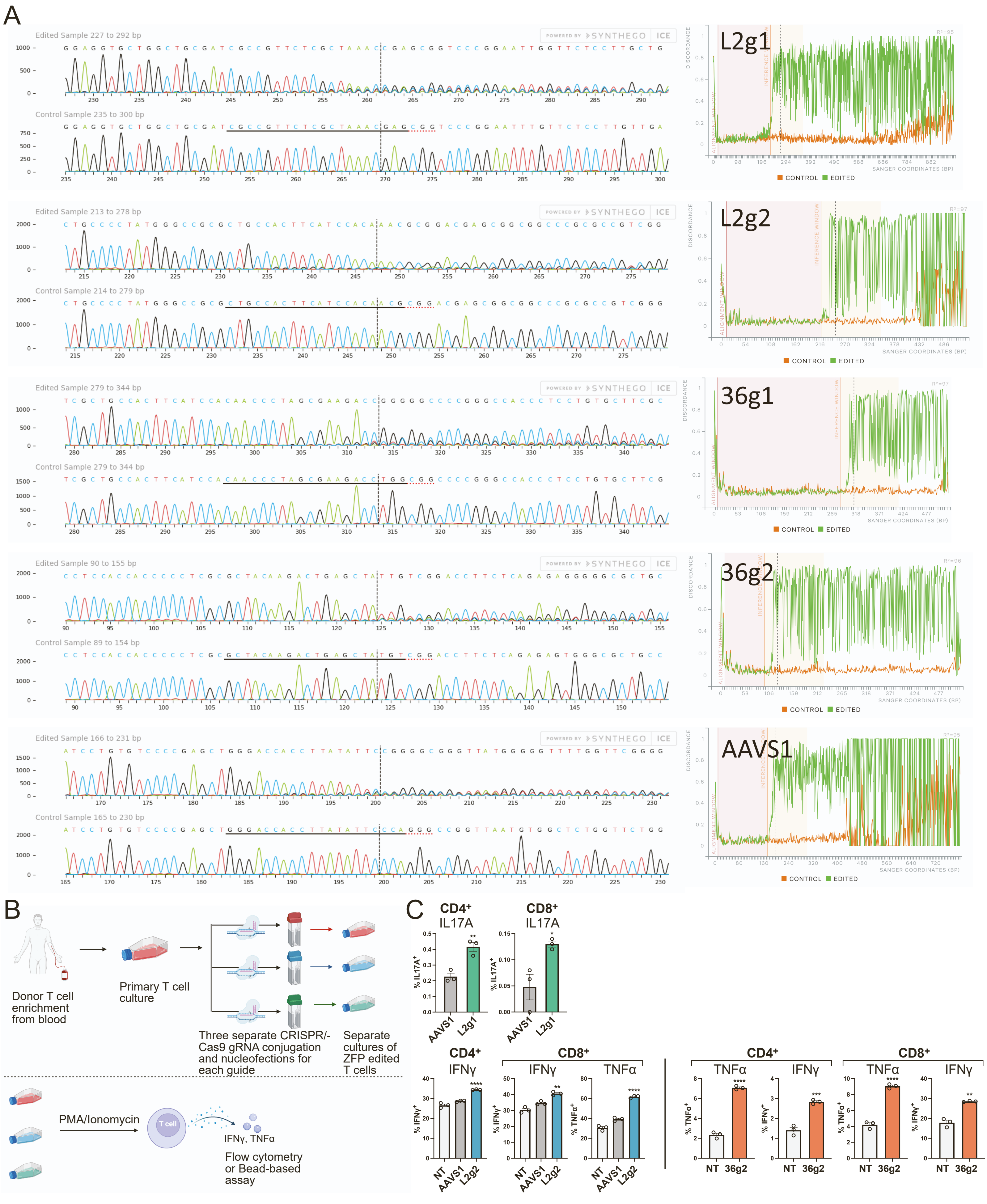

Figure S5 - Sanger sequencing details on CRISPR/Cas9 guides and additional flow cytometry data for a distinct donor (Related to Figure 3). A) Representative Sanger traces confirming CRISPR/Cas9 knockdown of guides used for Figure 3. B) Schematic showing CRISPR/Cas9 knockout strategy in Figure 3. C) Additional CRISPR/Cas9 knockout experiments and intracellular cytokine staining results for IL17A (2nd guide displayed), IFNγ and TNFα in *ZFP36L2* knockout cells, and IFNγ and TNFα in *ZFP36* knockout cells.

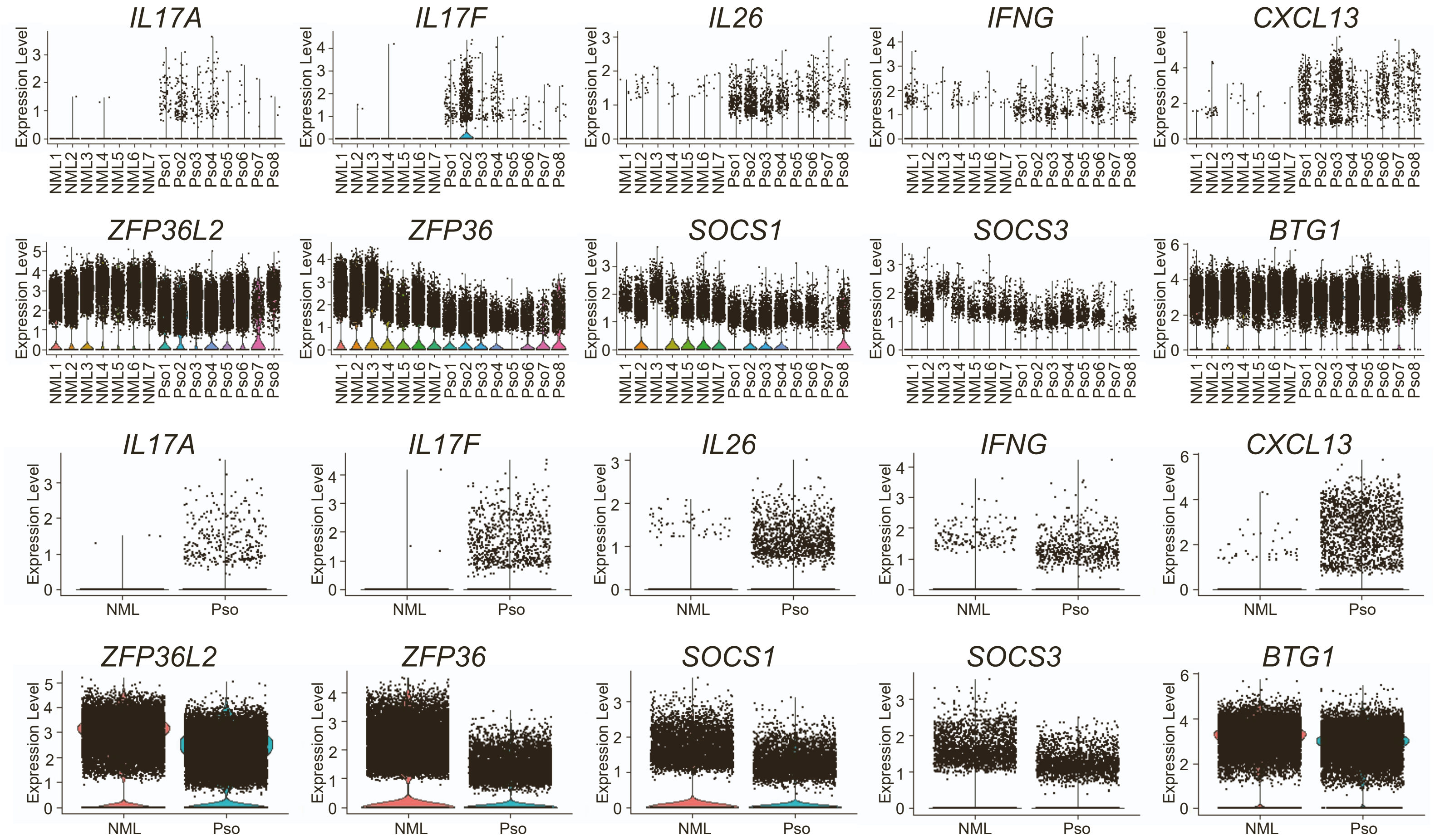

Figure S6 – Individual and aggregate patient expression of representative *ZIST* and Th17/Tc17 genes, showing sample-level consistency in DEG patterns (Related to Figure 5). Violin expression plots demonstrating consistency of expression changes in key *ZIST* and Th17/Tc17 inflammatory transcripts across individual and aggregated samples.
